# Unraveling the complexity of the histone code: implications for gene regulation and disease

**DOI:** 10.1186/s13059-026-04011-3

**Published:** 2026-03-24

**Authors:** Kiera L. Malone, Ajit K. Singh, James M. Lignos, Elizabeth D. Cook, Margaret Phillips, Brian W. Boyle, Isabelle A. Kressy, Mirabella Vulikh, Annika K. Lathrop, Kyle T. McKay, Hassan Zafar, Janet L. Stein, Gary S. Stein, Karen C. Glass

**Affiliations:** 1 Department of Pharmacology, Larner College of Medicine, University of Vermont, Burlington, VT 05405 USA; 2 Department of Biochemistry, Larner College of Medicine, University of Vermont, Burlington, VT 05405 USA; 3 University of Vermont Cancer Center, University of Vermont, Burlington, VT 05405 USA

**Keywords:** Epigenetics, Histone code, Histone post-translational modifications (PTMs), Histone crosstalk, Nucleosomes, Writers, Readers, And erasers, Histone tails, Combinatorial histone modifications, Chromatin architecture, Chromatin remodeling, Epigenetic regulatory mechanisms, Epigenetic dysregulation, Chromatin dynamics, Histone-binding domains, Transcriptional regulation through chromatin

## Abstract

Histone post-translational modifications epigenetically regulate gene expression by modulating nucleosome structure and DNA accessibility. This review outlines how unique patterns of modifications on canonical and variant histones generate a combinatorial histone code. Interplay between multiple modifications increases the complexity as distinct histone proteoforms and nucleoforms can drive context-dependent outcomes. The histone code is highly dynamic, and we examine how histone modifications fluctuate with environmental cues, metabolic state, and cell-cycle progression. Finally, we discuss how dysregulation of histone modifications contributes to disease, and how emerging technologies to decipher the histone code are yielding new biological insights and opportunities for therapeutic development.

**Supplementary Information:**

The online version contains supplementary material available at 10.1186/s13059-026-04011-3.

## Introduction

**Fig. 1:**
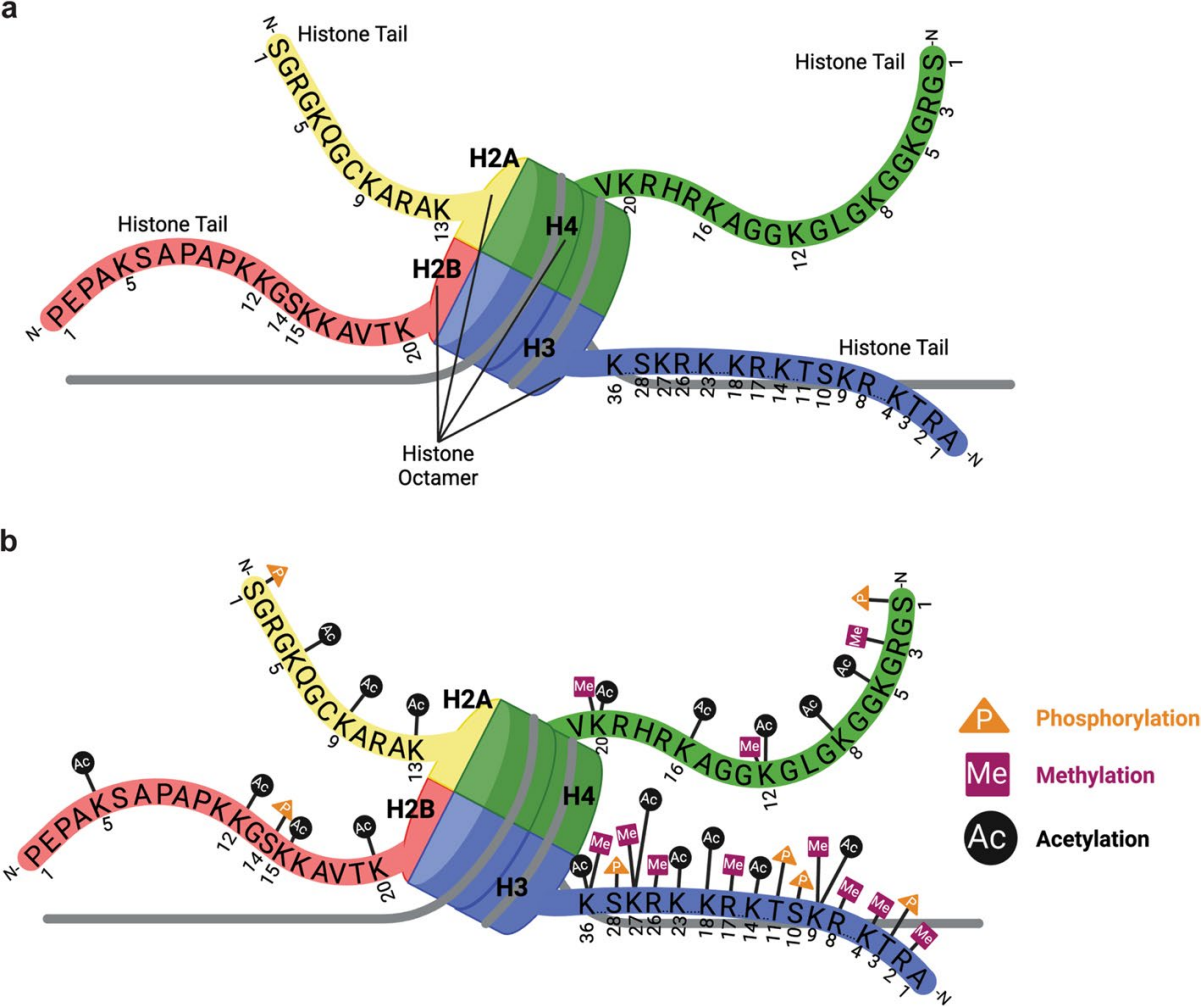
Structural organization and post-translational modifications of the nucleosome core particle. **a** The nucleosome core particle is formed by two copies of each of the four canonical histone proteins- H3 (blue), H4 (green), H2A (yellow), and H2B (red)- that create an octamer, around which approximately 147 bp of double stranded DNA is wrapped. The protruding tails (one tail shown for each histone protein for clarity) of each histone protein are colored to match their respective globular units. The amino acid residues are represented with their single letter codes and are numbered according to the N-terminus of the histone proteins. **b** The N-terminal tails exhibit post-translational modifications, denoted by orange triangles for phosphorylation, pink squares for methylation, and black circles for acetylation

The concept of “epigenetics” was first introduced in 1942 when Conrad Waddington described the idea of trying to determine how genotypes and phenotypes are connected as the “epigenotype” [1]. He surmised that a general feature of the epigenotype, “…consists of concatenations of processes linked together in a network” [1]. Epigenetics is now a vast field of study aimed at understanding the mechanism and modifications which alter gene expression without making changes to the underlying DNA. The human genome is nearly three meters long and needs to be arranged in such a way so that it can fit into the < 10 µm wide diameter of the nucleus while remaining easily accessible [2]. The compaction of proteins and DNA into a tight complex, called chromatin, plays a fundamental role in achieving this feat in eukaryotes. The main protein component of chromatin is composed of four canonical histone proteins: H2A, H2B, H3, and H4 [3]. Generally, two copies of histone H3 and H4 assemble into a tetrameric complex that is further organized into a histone octamer by the addition of two heterodimers of histones H2A/H2B [4]. In addition to the canonical histone proteins, the human genome also encodes histone variants, which differ from the canonical histones only by a few amino acids. At the N-terminus of each canonical and variant histone protein is an unstructured, flexible extension known as the “histone tail”, which protrudes from the histone octamer (Fig. 1a). While expression of histone variants is regulated similarly to the canonical core histones, they often have niche cellular functions. Due to their high lysine and arginine abundance, histone proteins carry a strong positive charge which assists in the ability to efficiently incorporate approximately 147 base pairs of negatively charged DNA around the histone octamer to form the nucleosome core particle (NCP) [3, 5, 6]. The NCP is considered the fundamental unit of chromatin [5], and is positioned throughout the genome with a spacing of approximately 200 DNA base pairs. The assembly of these “beads on a string” is the first step in the chromatin compaction process that forms higher order chromatin structures, such as chromatin fibers and mitotic chromosomes [7]. Within a chromosome, there are highly condensed regions known as heterochromatin where multiple consecutive repeats of nucleosomes compact DNA and silence transcription. Conversely, euchromatin regions contain DNA that is less constricted and more amenable to being accessible to transcription factors for gene expression [2, 5, 8]. While we are aware of these different types of chromatin structures, how the genetic information contained within them is remodeled to regulate gene expression is an active area of investigation. Here we review the basis for histone code-mediated transcriptional competency of gene loci, which is intricately tied to the higher-order organizational structures of chromatin.

To date, three epigenetic mechanisms have been identified to solve the challenge of organizing the complex eukaryotic genome while ensuring access for gene expression: DNA methylation, non-coding RNA (ncRNA)-associated gene silencing, and histone modifications [9]. The N-terminal tails of both canonical and variant histone proteins are rich in amino acid residues that are subject to post-translational modifications (PTMs) [10]. Histone PTMs alter the chemical properties and conformation of histone proteins, thereby influencing how nucleosomes interact with DNA and regulatory factors. These modifications involve the covalent addition of small chemical groups, most prominently phosphorylation, methylation, and a diverse set of acylations [10]. Acyl groups contain a carbonyl linked to variable side chains, and the simplest and most abundant example is the acetyl group, which neutralizes the positive charge on lysine residues and loosens histone-DNA interactions. Beyond acetylation, several other acylations have been identified (Figure S1), including propionylation and butyrylation, which introduce longer aliphatic chains and subtly alter histone–chromatin contacts. More recently, crotonylation was discovered, and unlike other acyl groups, the crotonyl moiety contains a carbon–carbon double bond that renders it planar and rigid, imparting unique structural and functional consequences [11, 12]. Methylation of DNA usually results in the formation of heterochromatin by recruiting gene suppressing protein effectors to silence gene transcription [9]. Non-coding RNA (ncRNA) molecules are created from DNA that is transcribed into functional RNA but are not translated to proteins. Examples of ncRNA include microRNAs (miRNA), short interfering RNAs (siRNA), and long non-coding RNAs (lncRNA). Growing evidence suggests ncRNAs are important for the regulation of gene expression by promoting heterochromatin formation to silence genes, much like DNA methylation [9]. Excellent reviews provide more information on epigenetic regulation via DNA methylation [13–17] and ncRNAs [18–22]. Our main focus here will be to review our current understanding of epigenetic regulation via post-translational modification of histones. Histone PTMs are highly responsive to environmental cues, reversible, and capable of controlling chromatin structure, cellular plasticity, and inheritance. They have evolved to regulate a vast number of gene regulatory networks, which provides a rich area of study.

## Histone post-translational modifications

### Individual histone modifications signal for specific functions

**Fig. 2:**
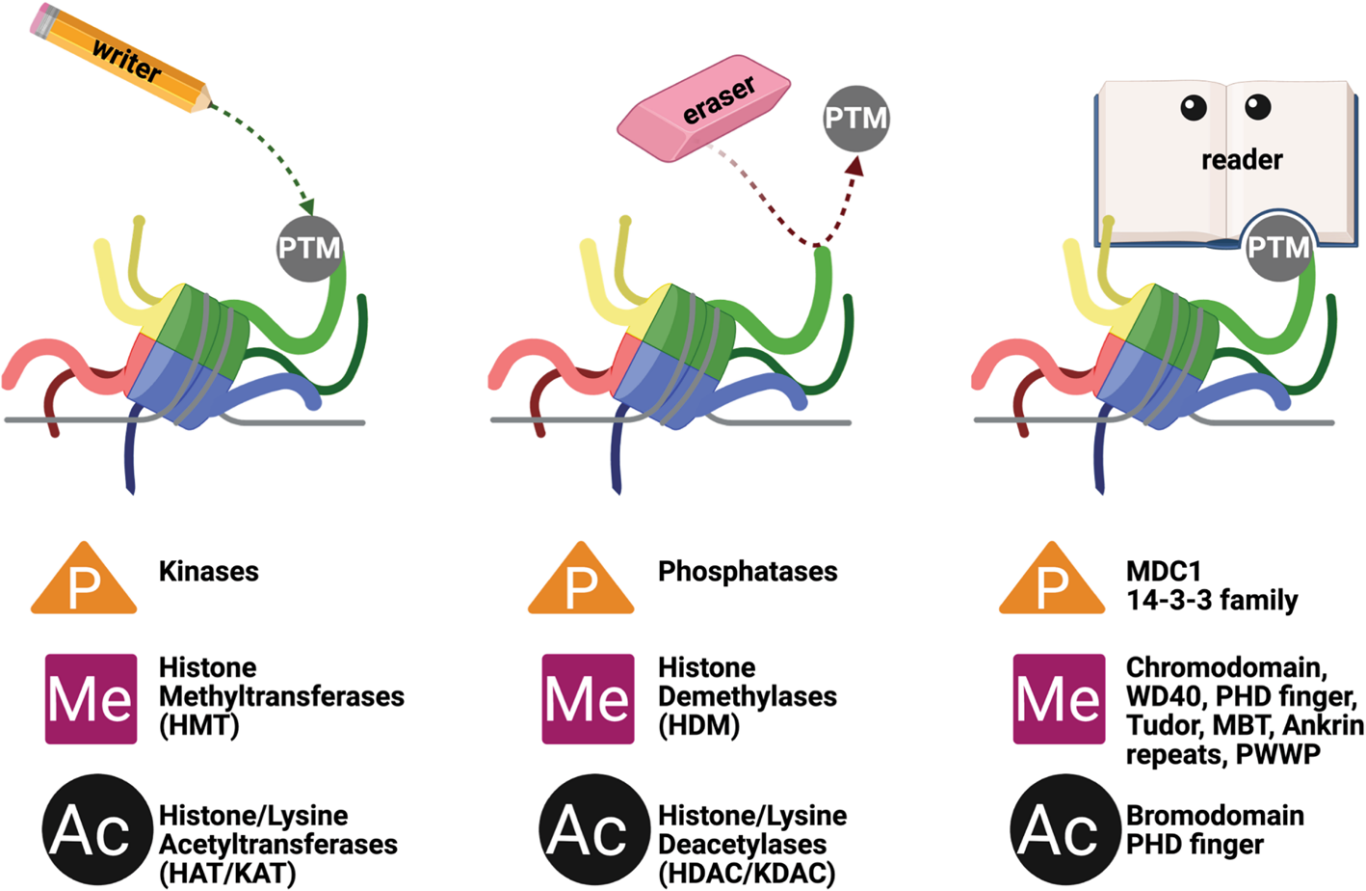
Three classes of proteins regulate the addition, removal, and interpretation of PTMs. Writer enzymes (left) add PTMs to histone proteins. Eraser enzymes (middle) remove PTMs from histone proteins. Reader proteins (right) recognize specific modifications and interpret the epigenetic signals contained within them. Included below each protein class are examples of proteins involved with certain modifications, with orange triangles representing phosphorylation, pink squares for methylation, and black circles for acetylation

The addition and removal of histone PTMs is reversible, and their composition on any given histone changes dynamically throughout the cell cycle, and in response to environmental cues [10, 23–25]. Protein families grouped into reader, writer, and erasers, are known to affect the presence of these PTMs, and some examples are provided in Fig. 2 [26–34]. Importantly, the presence of single and multiple modifications on the histones form a “histone code,” and different combinations of these PTMs are linked to distinct cellular activities and phenotypical outcomes [35]. Phosphorylation, acetylation, methylation, and ubiquitination stand out as the most common and extensively studied among over 300 post-translational modifications identified [36, 37]. As outlined in more detail below, these four modifications exert influence across all facets of biological process regulation. These “above the gene,” or epigenetic regulatory signals often play crucial roles in cellular functions by recruiting multiprotein complexes, including chromatin remodelers, transcription factors, and DNA repair enzymes, to activate or inhibit various cellular signaling pathways [37].

**Fig. 3:**
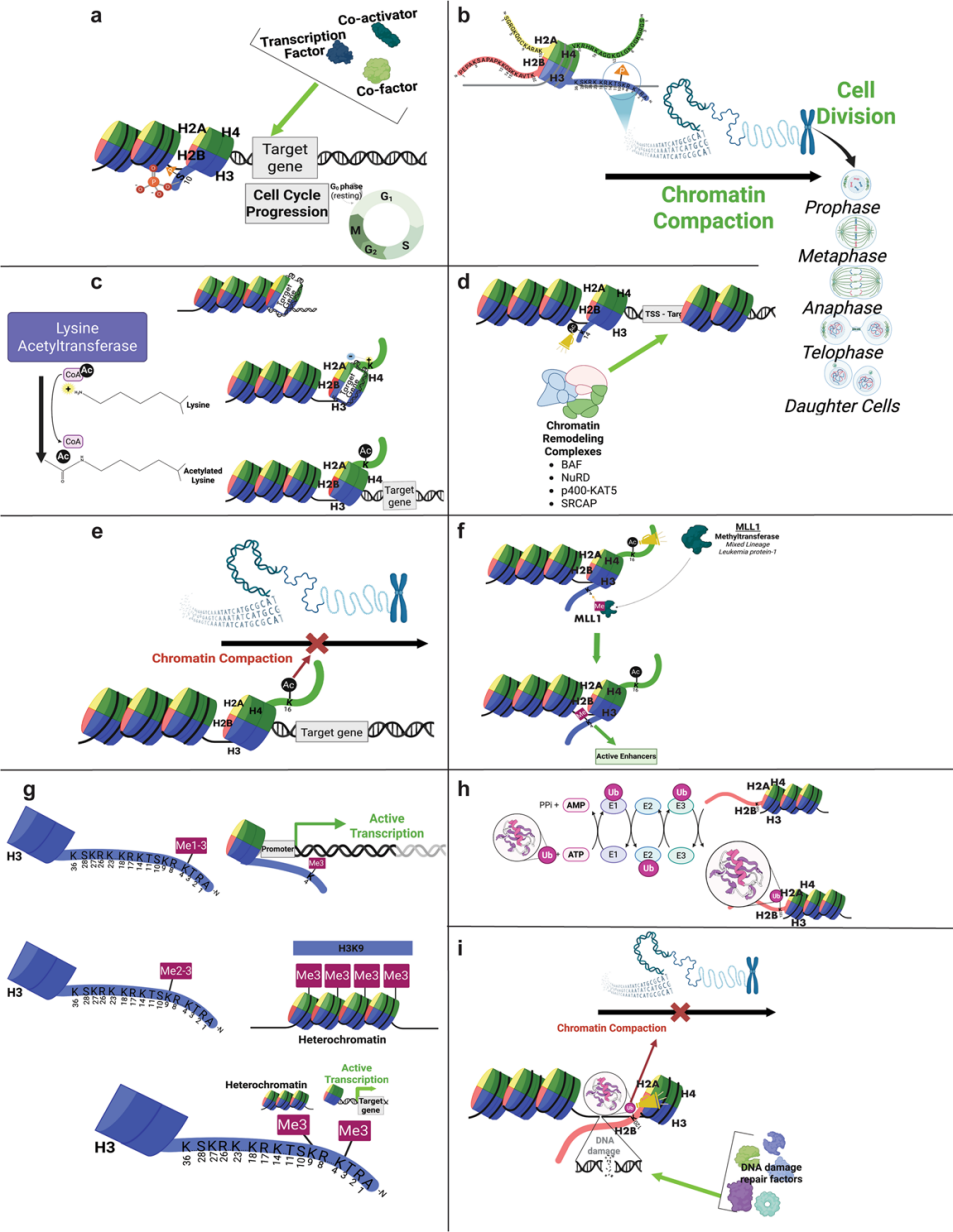
Functions of histone post-translational modifications. **a** Histone H3S10 phosphorylation promotes chromatin relaxation and gene activation. When histone H3S10 is phosphorylated the phosphate group’s charge repulsion forces can destabilize the compact nucleosome structure and decondense chromatin [41]. Histone H3S10ph is also known to recruit transcription factors and co-activator proteins that activate gene transcription and cell cycle progression [42, 43]. **b** Histone H3S10 phosphorylation drives chromatin condensation during mitosis. Histone H3S10ph is an important mark required for preparation of cell division in meiosis and mitosis [44]. During metaphase, extensive H3S10ph signals chromatin compaction for mitotic entry [45]. **c** Acetylation weakens histone-DNA interactions. When an acetyl group is transferred from the acetyl-CoA co-factor onto lysine and arginine residues on histone proteins it neutralizes their positive charge and subsequently weakens the interactions between the DNA and histones within the nucleosome [55]. This destabilization of the nucleosome core particle increases the accessibility of DNA for transcription [56]. **d** Acetylation modifications recruit chromatin remodelers. Acetylation of histone H3 at lysine 14 (H3K14ac) recruits chromatin remodeling complexes to change nucleosome spacing at transcriptional start sites (TSS) and facilitates DNA damage repair [57]. **e** Acetylation disrupts higher-order chromatin packing. Acetylation of H4K16 blocks inter-nucleosome contacts, preventing the formation of higher-order chromatin structures, and promotes active gene expression [58]. **f** Acetylation serves as a platform for effector proteins. Acetylation of histone H4K16 by the KAT8/MOF HAT recruits the MLL4 methyltransferase complex to write the histone H3K4me1 modification [59, 60]. **g** Histone methylation patterns define active and repressive chromatin states. Methylation of histone H3 at lysine 4 can occur in mono-, di-, and tri- methyl forms (H3K4me1, me2, and me3) and is closely linked with active transcription (top) [61–63]. Histone H3K4me1 serves as a marker for enhancers, while histone H3K4me2 is predominantly found surrounding actively transcribed genes [64]. Histone H3K4me3 is most frequently located at the promoters of transcriptionally active genes (top) [63]. Histone H3K9 di- and tri-methylations mark repressed genes, heterochromatin, and non-coding regions of the genome (middle) [65]. Despite their proximity on the histone H3 tail, the methylation state of K4 or K9 convey distinct epigenetic signals that drive opposing transcriptional states (bottom). **h** Sequential enzymatic steps of histone ubiquitination. First, the ubiquitin-activating enzyme (E1) binds ubiquitin, then the ubiquitin conjugating enzyme (E2) transfers ubiquitin from E1 to E3, and finally the ubiquitin ligating enzyme (E3) links ubiquitin to the lysine residue of a histone protein [66]. **i** Ubiquitination maintains open chromatin for DNA repair. Histone H2BK120 mono-ubiquitination promotes DNA double-strand break repair by maintaining open chromatin and coordinating recruitment of repair factors [67–69]

Histone phosphorylation is associated with chromatin remodeling for transcription regulation, recruiting repair machinery for the DNA damage response, and chromatin compaction during meiosis and mitosis [38]. The addition of a phosphate group to these polar amino acids introduces a negative charge and causes conformational changes that may alter enzyme activity or protein–protein interactions [39, 40]. As shown in Fig. 1b, phosphorylation occurs at several sites in the nucleosome core particle. The functional effects of these phosphate groups depends on which histone residue they are added to, and how the epigenetic signal will be interpreted by other cellular machinery and protein effectors [38]. As depicted in Fig. 3a-b, phosphorylation can activate gene expression via charge repulsion and recruitment of transcription factors [41–43], or it can trigger chromatin condensation for mitosis [44, 45]. Table S1a provides additional examples of phosphorylation modifications found on the four canonical histone proteins and the cellular functions that are associated with each mark [38, 46–49]. This information highlights the importance of deciphering how the histone code functions as a signaling platform to regulate essential cellular pathways. A key challenge in these studies is the promiscuity of kinases, which frequently target multiple substrates, complicating the attribution of specific phosphorylation events to discrete biological outcomes [50–52]. While studies employing kinase inhibitors and histone-tail mutants that prevent addition of phosphorylation modifications are informative, the complexity of kinase signaling cascades (characterized by extensive crosstalk and feedback loops) limits our ability to link individual histone phosphorylation marks to distinct physiological processes [52]. To overcome this, an integrated approach using multiple methods such as such as ChIP-seq, RNA seq, and quantitative mass spectrometry, combined with targeted perturbation and phenotypic assays, will be essential to elucidate the dynamic landscape of histone phosphorylation and its downstream effects [53, 54].

Histone acetylation has been well documented to have a role in the regulation of gene transcription, and the presence of acetylated histones results in increased transcription and gene activation [27, 70–74]. Additionally, many studies have shown that genomic regions containing acetylation modifications are generally associated with up-regulated gene expression (Table S1b) [35, 75, 76]. Figure 3c-f outlines how acetylation of histone proteins can alter gene expression patterns [55, 56, 58, 59]. Although histone acetylation is well studied in gene activation, many lysine acetyltransferases (KATs), such as p300/CBP, also acetylate non-histone proteins, complicating interpretation of specific physiological effects. For instance, p300/CBP-mediated histone H3K14 and H3K56 acetylation is linked to DNA damage repair [57, 77, 78], but these enzymes also acetylate non-histone targets such as p53 and Hypoxia-Inducible Factor 1-alpha (HIF-1α) [79, 80], which also function in DNA repair, confounding the specific role of histone acetylation.

Methylation of histones functions as an epigenetic signal that is associated with a wide variety of cellular processes such as the activation or repression of gene expression, the regulation of cell cycle stages and progression, and cellular responses to stress including DNA damage pathways (Fig. 3g and Table S1c) [61, 62, 65, 81, 82]. Multiple methyl groups can be added to the epsilon nitrogen of lysine, such that it is mono-, di-, or tri-methylated on a single residue, allowing the cell to fine-tune functional outcomes [10, 63, 64]. Similarly, up to two methyl groups can be added to the side chain of arginine residues, and they can occur in multiple orientations on the arginine guanidino group producing two different regioisomers [83–86]. Asymmetric methylation of arginine occurs when only one of the terminal nitrogen groups is di-methylated [85, 87]. Symmetric di-methylation occurs when one methyl group is added to each of the two terminal nitrogens in the arginine side chain [88, 89]. The multiplicity factor of epigenetic signaling through methylation provides an additional layer of control over transcriptional processes that can be used to dynamically regulate the interaction of chromatin regulatory complexes [90].

Histone ubiquitination plays a multifaceted role in maintaining genomic integrity and cellular homeostasis (Table S1d) [91–95]. As shown in Fig. 3h ubiquitination occurs through a selective and ATP-dependent mechanism that covalently attaches the 76-amino-acid ubiquitin polypeptide to the epsilon nitrogen of lysine residues via a peptide bond to the C-terminus of ubiquitin [66, 96]. Figure 3i outlines the role of histone ubiquitination in the DNA damage repair process, which highlights how it functions to modulate nucleosome dynamics for transcriptional regulation of essential biological functions [66–69, 96].

In addition to the modifications described above, cells contain a wide array of histone PTMs. A comprehensive SnapShot published in 2014 cataloged 15 distinct types [97]. Advances in technology have since uncovered several new histone modifications, and current research is focused on understanding their roles in the complexity and dynamics of the histone code. One emerging class of PTMs involves monoamine neurotransmitters such as serotonin and dopamine, which are added to histone H3 at Gln5 via transamidation. These modifications, known as serotonylation and dopaminylation, respectively, alter the flexible histone tail and appear to regulate neural transcription by reshaping global chromatin states [98]. The combination of histone H3Q5ser and H3K4me3 facilitates recruitment of transcriptional machinery, leading to elevated gene expression [99, 100]. The synergistic effect between serotonylation and methylation highlights the role of serotonylation in fine-tuning transcriptional responses. Histone H3Q5ser has also been implicated in cancer progression, including the pediatric brain tumor ependymoma, where it modulates the epigenetic landscape as an activating modification [101]. These findings establish a direct link between neurotransmitter signaling and gene regulation, revealing a novel mechanism of epigenetic control.

Histone modifications generate intricate signaling patterns that form a dynamic regulatory network governing a wide range of cellular processes. One limitation in deciphering their specific roles lies in the multifunctionality and substrate promiscuity of the modifying enzymes. Also, many studies linking histone modifications to cellular phenotypes are correlative. However, several mutations in key histone residues have been shown to phenocopy deletion of the corresponding modifying enzymes (Table S2), which validates the functional importance of specific histone modifications in regulating downstream pathways [102–104]. Moving forward, integrative approaches combining genomic, proteomic, and functional assays will be critical to unravel the causal relationships between specific histone modifications and downstream biological processes.

### Histone variants further expand the functionality of the histone code in epigenetic regulation

The modifications mentioned above are found on the four canonical histones: H2A, H2B, H3, and H4 (Fig. 1). There are also a number of histone variants encoded in the human genome that play essential roles in unique cellular functions [105]. Whereas the canonical histone proteins are typically only generated once the cell commits to dividing, the histone variants are often expressed throughout the cell cycle [106]. Just like the canonical core histones, the histone variants are subjected to a variety of PTMs [107, 108]. The amino acid sequence of histone variants is divergent, and these differences facilitate their specialized roles in various cellular processes including chromatin organization, DNA damage repair, mitosis, and transcriptional activation/repression. For example, the histone variant H2A.X plays a central role in DNA double-strand break (DSB) repair [109]. Upon damage, histone H2A.X is phosphorylated at serine 139 (H2A.XS139ph or γH2A.X), creating a signal that recruits DNA repair enzymes, which then initiate repair through homologous recombination or non-homologous end joining [110].

Another example is histone H3.3 which differs from its replication-associated counterpart histone H3 by only five amino acids [111, 112]. However, these sequence variations significantly influence its function, and one notable distinction is the substitution of Histone H3.1Ala31 with a serine residue. Phosphorylation of Ser31 on histone H3.3 (H3.3S31ph) emerges as a pivotal marker during mitosis, frequently observed in proximity to centromeres during prometaphase/metaphase [113]. Despite its prevalence, the precise role of H3.3S31ph in mitosis remains elusive. Interestingly, more recent studies by Armache et al. discovered that when H3.3 Ser31 is phosphorylated it rapidly activates stimulation-induced genes by blocking the interaction of the ZMYND11 transcriptional corepressor, and recruits the SETD2 H3K36 methyltransferase to increase accessibility of the transcriptional machinery [43].

Although histone variants add complexity and fine-tune the regulation of diverse cellular processes, several limitations are hindering our progress to fully understanding their functions. For example, the histone variants are present at much lower levels than canonical core histones, making it challenging to define their specific functions and genomic distributions using chromatin immunoprecipitation sequencing (ChIP-seq) [114]. Additionally, histones and their variants often differ by only by a few amino acids, making it difficult to distinguish them and their associated post-translational modifications using antibody-based methods and mass spectrometry. In summary, the diverse array of histone variants further expands the histone code through their unique amino acid sequences that provide additional sites for modifications and specialized functions. Their presence underscores the intricate regulation of chromatin dynamics, and the function of histone variants in epigenetic regulation of cellular processes is an ongoing area of investigation.

## Combinations of histone modifications increase the complexity of the histone code

The “histone code” hypothesis spurred investigation into how combinations of histone PTMs collectively influence chromatin structure and gene expression [35, 115]. Although individual modifications can recruit effector proteins, it is now evident that their combined impact determines overall chromatin states and provides an additional layer of regulation. The combinatorial diversity generated by multiple modifications exponentially amplifies the complexity of the histone code, leading to the concept of the ‘histone proteoform’, which refers to the unique pattern of PTMs found on an individual histone protein [116]. Over 200 histone proteoforms have been identified [117], yet their specific functional roles remain largely undefined. Additionally, the distinct composition of PTM histone proteins within a single nucleosome is known as the nucleoform [118], and distinguishing between descrete PTMs, proteoforms, nucleoforms, and experimental settings where the context is unclear necessitates a well-defined and descriptive nomenclature [119]. Adding to the complexity, these modification patterns are dynamic, and change in response to the cell cycle progression [120–122], disease states [123, 124], diet, and environmental stimuli [125]. Despite some known examples of PTM crosstalk, the full extent of interactions within and between histone proteoforms remains poorly understood. The immense number of possible combinations of PTM histones, proteoforms, and nucleoforms poses a major challenge to decoding the histone code. The following section highlights key examples underlying the complexities of this epigenetic regulatory mechanism.

### Crosstalk among histone modifications- acetylation and phosphorylation interactions

Acetylation and phosphorylation on histone H3 were first observed together following epidermal growth factor stimulation in mouse 10T1/2 cells [126]. Phosphorylation of histone H3S10 or H3T11 promotes acetylation on histone H3K9 and H3K14 by recruiting the histone lysine acetyltransferase 2 A (KAT2A, also known as general control non-derepressible 5, GCN5) which promotes gene transcription [126]. Kinetic studies show that GCN5 preferentially acetylates histone H3 peptides phosphorylated at Ser10, indicating that phosphorylation precedes and facilitates acetylation [126]. Consequently, this modification cascade correlates with enhanced transcriptional activity [38]. Interestingly, the interplay between phosphorylation and acetylation PTMs can also facilitate chromatin compaction. Histone H3S10ph, in the presence of histone H4K16ac, recruits the histone deacetylase Hst2p (a yeast homolog of the human SIRT2 protein), which removes the acetyl group at H4K16ac and enables the histone H4 tail to interact with the H2A-H2B dimer interface of a neighboring nucleosome, promoting chromatin condensation [45]. These examples illustrate how crosstalk between phosphorylation and acetylation modifications can yield opposing outcomes depending on context, highlighting the regulatory complexity of combinatorial histone PTMs.

### Crosstalk among histone modifications- methylation and ubiquitination interactions

Interactions between methylation and ubiquitination also play significant roles in regulating gene expression. While histone H3K4me3 is generally associated with transcriptional activation (Table S1c) [61, 127], mono-ubiquitination of histone H2A can inhibit the transition from H3K4me2 to H3K4me3 within the same nucleosome, thereby repressing transcription [128]. In *Saccharomyces cerevisiae*, histone H2BK123ub is required for proper methylation of histone H3K4 by the COMPASS/Set1 (complex proteins associated with Set1) complex [73, 129–133]. This ubiquitin mark facilitates recruitment of Set1, the sole methyltransferase for histone H3K4 in yeast, and also promotes H3K79 methylation. Notably, in yeast, histone H3K4me3 can act as a repressive signal under stress conditions [129, 130, 134]. This gene repressive function of histone H3K4me3 in yeast is different from its well-established role in promoting gene transcription in humans [135]. This highlights how the same PTM can mediate distinct regulatory outcomes across species. Moreover, proximal PTMs can disrupt the enzymatic activity of Set1 and other modifying enzymes, adding another layer of complexity to transcriptional control. These examples underscore the context-dependent nature of PTM crosstalk and the evolutionary diversity of epigenetic regulation.

### Crosstalk among histone modifications- phosphorylation and methylation interactions

The interplay between histone modifications plays a pivotal role in coordinating gene expression and cell cycle progression. A well-characterized example involves histone H3K9me3, which recruits heterochromatin protein 1 (HP1) to establish transcriptionally repressive heterochromatin [65]. This interaction between HP1 and histone H3K9me3 is dynamic, as HP1 is present during interphase but largely lost in M phase [136, 137]. Mass spectrometry analysis of HeLa cells revealed that phosphorylation at histone H3S10 occurs adjacent to H3K9me3 specifically in mitotic chromatin, correlating with HP1 dissociation [137]. Peptide-binding assays confirmed that histone H3S10ph significantly reduces HP1 affinity for H3K9me3, suggesting that phosphorylation serves as a reversible switch to disrupt heterochromatin during mitosis. Aurora B kinase further enhances this effect, displacing HP1 from chromatin and promoting chromosome condensation [137].

In contrast, histone H3S10ph also acts as a transcriptional activator in certain contexts. The serine/threonine kinase Pim-1 phosphorylates H3S10 at the *FOSL1* enhancer upon serum stimulation, initiating a cascade that recruits the 14-3-3 reader protein and KAT8/MOF (lysine acetyltransferase 8/males absent on the first), leading to histone H3K14 acetylation [138]. This modification promotes bromodomain-containing protein 4 (BRD4) binding to histone H3K14ac, which facilitates release of paused RNA polymerase II and triggers transcription elongation [139].

Together, these examples illustrate how the H3S10ph modification can either disrupt repressive chromatin or initiate transcription, depending on its context and neighboring PTMs [137, 138]. This dual functionality highlights the importance of understanding how PTM crosstalk recruits effector proteins and fine-tunes chromatin states to regulate gene expression.

### Crosstalk among histone modifications- methylation and acetylation interactions

Histone methylation and acetylation predominantly targets lysine and arginine residues, and often compete for the same lysine sites. For example, histone H3 can undergo acetylation or methylation at positions K9, K27, and K36 (Fig. 1b). This implies that one modification may inhibit the other, but histones frequently harbor a mix of methylation and acetylation, showcasing the complexity of epigenetic regulation. Marunde et al. explored this interplay by studying BPTF (Bromodomain PHD finger transcription factor), a dual reader protein containing a PHD finger that recognizes histone H3K4me3 and a bromodomain that binds acetylated lysines [30, 135, 140]. Using combinatorial arrays of modified histone peptides and recombinant nucleosomes, they found BPTF has the strongest affinity for histone H3K4 mono-, di- and tri-methylated histone peptides [140]. However, the binding of BPTF PHD-bromodomain to nucleosomes containing histone H3K4me3 was over 80-fold weaker than to free peptides. Notably, the interaction between BPTF and nucleosomes was significantly enhanced when acetylation was present alongside methylation on the same histone protein (specifically H3K4me3K14ac or H3K4me3K18ac). Acetylation likely weakens histone-DNA contacts via charge repulsion, increasing tail accessibility and enabling the PHD-bromodomain module to recognize dual modifications more effectively [140]. Further validation using a reader CUT&RUN assay revealed that BPTF is enriched at genomic sites with co-occurring histone H3K4me3 and H3K18ac marks, supporting a model in which the synergistic effects of methylation and acetylation enhance reader recruitment and promote downstream chromatin remodeling [140].

### Crosstalk among histone modifications- interplay between homotypic histone modifications

Methylation at histone H3K4 is a hallmark of active transcription, yet nearby modifications can strongly influence its establishment. WD40-repeat protein 5 (WDR5), a subunit of the mixed lineage leukemia 1 (MLL1) methyltransferase complex, binds histone H3K4me2 and facilitates its conversion to H3K4me3 [141]. Methylation at histone H3R2 disrupts WDR5 binding, blocking this conversion [142]. Hyllus et al. showed that while MLL1 efficiently methylates unmodified histone H3K4, its catalytic activity is modulated by the methylation status of H3R2 [143]. MLL1 methylation of histone H3K4 was impaired in the presence of mono-methylated H3R2, and strongly inhibited by H3R2me2 [143]. The results demonstrate that modifications adjacent to histone H3K4 can influence its methylation status and, consequently, the activation of gene transcription. The activity of protein effectors can further modulate the interplay between histone modifications. PRMT6 (protein arginine methyltransferase 6) is the main methyltransferase for histone H3R2 in humans [143]. Overexpression of PRMT6 in MCF7 breast cancer cells suppresses histone H3K4me3 [142], whereas PRMT6 knockdown enhances it [143]. Collectively, these findings confirm that methylation of histone H3R2 by PRMT6 negatively regulates H3K4me2 methylation by the MLL1 complex.

Acetylation patterns also exhibit homotypic coordination. On histone H4, acetylation proceeds in a sequential manner from K16 to K12, K8, and K5 in a “zip model” [144, 145]. This culminates in a hyperacetylated state of histone H4, and deacetylation follows the reverse order [144, 146]. On histone H3, acetylations are added in a more flexible sequence starting at H3K14, followed by K23, and then K18, while K9 acetylation appears to be independently regulated [145]. Histone H2B also displays a preference for acetylation sequence, with K12 and K15 typically acetylated before the K5 and K20 residues [145].

The mechanistic rationale for ordered acetylation remains unclear, but acetylation reduces histone tail-DNA interactions via charge repulsion, increasing chromatin accessibility [140]. Hyperacetylation of histone H4 enhances the flexibility and accessibility of the histone H3 tail [147], facilitating the engagement of chromatin regulators such as MLL1 and WDR5 to promote H3K4 methylation [148]. Hyperacetylation of histones H3 and H4 has long been associated with euchromatin and regions of active transcription [149]. In addition, hyperacetylated histones serve as binding platforms for specific chromatin remodeling complexes. For example, the PBAF and BAF subunits of SWI/SNF preferentially bind nucleosomes with multiply acetylated histone H3, while INO80 and CHRAC favor nucleosomes with hyperacetylated H4, and the NUA4 complex recognizes nucleosomes carrying acetyl marks on both histone H3 and H4 [150].

Chromatin regulatory proteins also show specificity for particular acetylation patterns. ATPase family AAA + domain-containing protein 2 (ATAD2) and its conserved paralog, ATAD2B, recognize acetylated lysines through their bromodomain regions. A combinatorial screen of multiple PTMs reveals the ATAD2 bromodomain binds only 11 unique histone ligands, while the ATAD2B bromodomain recognizes 39, suggesting it has a broader specificity [151]. Furthermore**,** particular combinations of histone modifications can influence recognition [152]. ATAD2 and ATAD2B favor histone H4K5acK12ac ligands, but nearby PTMs can alter their binding interaction (Figure S2) [153]. These preferences likely reflect distinct cell cycle functions as ATAD2 is predominantly expressed in S-phase where it protects newly synthesized histones (e.g., H4K5acK12ac) from deacetylation by histone deacetylase 1 (HDAC1) and HDAC2 until they are assembled into chromatin in preparation for cell division [122, 154]. ATAD2B may act at other stages when histone variants are incorporated [155]. BRD4 provides another example of bromodomain selectivity. Its two bromodomains, BD1 and BD2, bind multiple histone H3 acetylation modifications [156], but show different affinities for acetylated histone H4 ligands due to sequence variation and the local amino acid environment surrounding acetyl-lysines [157]. These findings suggest that different acetylation patterns act as a regulatory mechanism to fine-tune subsets of active chromatin states and their recognition by protein effectors.

### Crosstalk among histone modifications- interplay between heterotypic histone modifications

Histones frequently contain multiple PTMs, and crosstalk between different types of modifications determines chromatin effector binding [158]. PTMs located near acetylated lysines can modulate bromodomain binding activity via changes in steric hindrance or charge. For example, the second bromodomain of BRD4 binds strongly to histone H3K4acK9ac, but this interaction is disrupted by tri-methylation of H3K9 [156]. Similarly, the ATAD2 and ATAD2B bromodomains display different sensitivity to nearby modifications (Figure S2) [153]. Furthermore, the bromodomain and PHD finger containing protein 1 (BRPF1) subunit of the KAT6A/MOZ (lysine acetyltransferase 6A/monocytic leukemia zinc finger protein) complex binds to histone H4K5ac, facilitating acetylation of particular chromatin regions [159, 160]. Phosphorylation of histone H4S1 adjacent to H4K5ac reduces the binding affinity of BRPF1 nearly threefold, from 79 µM to 220 µM. This result suggests that phosphorylation of histone H4S1 likely functions as a signal to switch from acetylation-associated transcriptional processes to recruitment of DNA damage response machinery [161]. These examples illustrate how combinatorial modifications impact bromodomain binding and provides a framework for understanding the importance of multiple PTMs on the regulation chromatin interactions.

Additionally, the interconnected functions of histone PTMs also mediate cellular processes. Histone modifications do not typically function as isolated marks, but as part of a coordinated network of PTMs that regulate chromatin states and gene expression. Through both cooperative and antagonistic interactions, different variations of histone proteoforms influence the recruitment and activity of chromatin-associated proteins, ultimately shaping cellular phenotypes. While mass spectrometry has enabled detailed profiling of PTMs on canonical and variant histones, challenges remain in resolving complex modification patterns. Histone tails often carry multiple PTMs simultaneously and some modifications have identical masses, which complicates interpretation of the mass spectrometry data making it difficult to differentiate between modifications [162, 163]. To address these challenges and better resolve combinatorial PTMs, approaches such as top-down MS to analyze intact histones, or protease digestion strategies that yield longer peptides are essential. Utilization of state-of-the-art mass spectrometers and MS analysis approaches including Fourier-Transform Ion Cyclotron Resonance Mass Spectrometry (FT-ICR MS) that provide high sensitivity, resolution, and accuracy, combined with quantitative techniques including multiplexed isobaric tagging technology for relative quantitation (iTRAQ) and stable isotope labeling by amino acids in cell culture (SILAC), offer powerful solutions to dissect the complexities of the histone code [164–167].

## Dynamics of the histone code

Decoding the static catalog of histone modifications provides only a partial view of chromatin regulation. The histone code is highly dynamic, continually written, erased, and interpreted in response to intrinsic and extrinsic signals. Cell cycle progression, nutrient availability, metabolic state, stress, and differentiation cues drive rapid and reversible PTM changes that adapt chromatin states to biological demands [168]. This section highlights how temporal and context-dependent PTM regulation allows chromatin to integrate environmental inputs and orchestrate gene expression.

### Nutrient availability and histone modifications

Nutrient availability is a central determinant of histone modifications and profoundly influences the epigenetic landscape [125, 169–171]. Histone acetylation provides one of the most direct examples of this coupling. Lysine acetyltransferases (KATs) require acetyl-CoA as a substrate to acetylate lysine residues on histone tails [172]. Consumption of carbohydrates, together with metabolic flux through pathways such as glycolysis and fatty acid oxidation, regulates cellular acetyl-CoA levels [172, 173]. HeLa cells cultured with only one of these carbon sources (e.g. glucose, glutamine, pyruvate, or certain amino acids) are able to sustain acetyl-CoA production and maintain global histone acetylation. In contrast, depletion of these precursors reduces acetyl-CoA pools and diminishes histone acetylation levels [174]. Interestingly, removal of vitamins or salts does not significantly affect global acetylation, highlighting that carbon substrates (glucose, glutamine, pyruvate, amino acids) are the key determinants of acetyl-CoA pools and global histone acetylation [174]. S-adenosylmethionine (SAM) is synthesized from methionine, folate, choline, and betaine. Deficiencies in these nutrients reduce SAM levels and alter histone methylation patterns, with downstream effects on gene expression and cellular differentiation [175, 176]. On the other hand, removal of methyl groups by Jumonji-C histone demethylases (JHDMs) requires α-ketoglutarate (α-KG), an intermediate of the tricarboxylic acid cycle, as well as Fe^2^⁺ and molecular oxygen [177]. α-KG can be generated endogenously from glucose or glutamine, but can also be supplied from the diet [178]. Its availability is variable: supplementation with vitamin C increases the efficiency of JHDMs by maintaining Fe^2^⁺ in its reduced form, while iron deficiency limits activity [179–181]. Importantly, α-KG levels decline with age, and reduced demethylation capacity has been proposed to contribute to age-associated epigenetic drift [181]. Methylation status can influence acetylation. For instance, H3K9 methylation promotes heterochromatin that resists acetylation, while fluctuations in SAM and α-KG levels link nutritional state to multiple layers of chromatin regulation.

Histone phosphorylation is also regulated by nutrient availability. For example, high glucose levels drive the insulin and insulin-like growth factor (IGF) signaling pathways. This activates two major kinase cascades, namely the phosphoinositide 3-kinase **-** protein kinase B (PI3K-AKT) pathway and the Ras-mitogen-activated protein kinase/extracellular signal-regulated kinase (Ras-MAPK/ERK) pathway [182, 183]. AKT, also known as protein kinase B, can phosphorylate histone H3 at threonine 45 to promote transcription termination [184]. Activation of Ras-MAPK/ERK results in rapid phosphorylation of histone H3 at serine 10 and/or serine 28 by the mitogen- and stress-activated kinases 1 and 2 (MSK1/2), which often promotes chromatin remodeling and transcription activation [41, 47, 185]. This illustrates how changes in nutrient availability are rapidly transmitted to regulate chromatin states via kinase signaling

Starvation provides a striking example of how metabolism reprograms histone modifications. During fasting, fatty acid breakdown generates ketone bodies, especially β-hydroxybutyrate. This metabolite modifies histones directly by inducing lysine β-hydroxybutyrylation (Figure S1) [186]. β-hydroxybutyrylation activates transcription of starvation-response genes, including those regulating fatty acid oxidation and gluconeogenesis. At the same time, β-hydroxybutyrate inhibits histone deacetylases and it can serve as an alternative substrate for acetyl-CoA in specific KAT reactions, thereby increasing histone acetylation [187]. The result is a coordinated transcriptional program that supports cellular adaptation to nutrient deprivation.

Nutrient availability influences histone modifications at multiple levels by regulating the supply of enzymatic substrates, by providing or restricting cofactors, by modulating the activity of modifying enzymes through dietary metabolites, and by activating signaling pathways that converge on chromatin. While much is known about how nutrient fluctuations affect the addition and removal of histone marks, far less is understood about how metabolite levels impact the recognition of these modifications by chromatin reader proteins. Addressing this question will be essential for clarifying how dietary inputs shape the histone code and influence susceptibility to disease.

### Histone modifications during the cell cycle

**Fig. 4:**
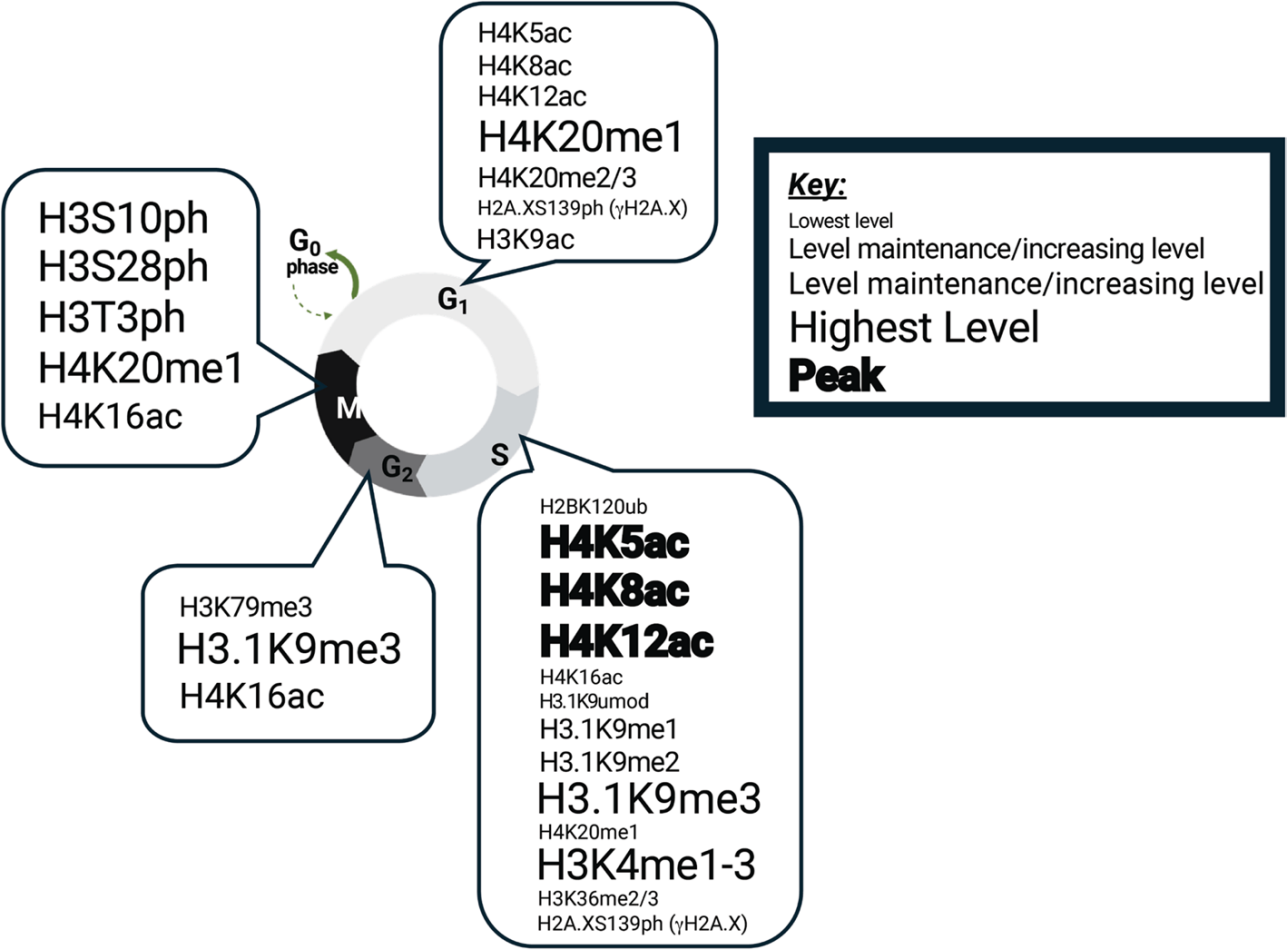
Dynamics of post-translational modifications across cell-cycle stages. The circular schematic shows G1 (white/light gray), S (light gray), G2 (gray), and M (black). PTMs are grouped by phase (Acetylation/ac, Methylation/me, Phosphorylation/ph, Ubiquitination/ub, or Unmodified/unmod), and font size approximates relative enrichment at that stage (larger and darker font = higher PTM levels), as shown in the accompanying key

Histone modifications change in phase-specific waves that coordinate the fundamental events of DNA replication, repair, and chromosome segregation) [121, 188, 189]. Although many PTMs remain constant, Fig. 4 summarizes the relative abundance (font size and color) for representative marks across the cell cycle phases G1 (white/light gray), S (light gray), G2 (gray), and M (black). Below, we track how the levels of several marks change in a phase-specific manner as depicted in the figure.

In early G1, cells grow and prepare for DNA replication. Histone modifications contribute to origin licensing and repair readiness. Histone H4 becomes acetylated at lysines 5, 8, and 12, and these modifications loosen chromatin packing near the replication origins [190]. Crosstalk with H4K20me1 is also critical as this mark recruits the KAT7/HBO1 acetyltransferase, which reinforces H4 acetylation and enables replication origin activation [190]. DNA repair pathways are also primed in G1. H4K20me2 recruits the p53-binding protein 1 (53BP1) to promote non-homologous end joining (NHEJ), the dominant repair pathway in this phase [191, 192]. Phosphorylation of H2A.X at serine 139 (γH2A.X) marks any double-strand breaks and recruits the mediator of DNA damage checkpoint 1 (MDC1) and breast cancer gene 1 (BRCA1) proteins, ensuring genome stability before DNA synthesis begins [193]. Acetylation of H3K9 at the cyclin D1 promoter facilitates transcription necessary for progression into S phase [194].

During S phase, a network of histone PTMs precisely regulates DNA replication to ensure genome stability. These modifications coordinate replication origin activation, fork progression, nucleosome disassembly and reassembly, and DNA repair. Ubiquitination of H2BK120 promotes replication origin activation and supports fork progression, while also participating in DNA repair [195, 196]. Acetylation of histone H4 at lysines 5, 8, and 12 significantly increases during S phase [190]. Newly synthesized histones carrying H4K5acK12ac are recognized by ATAD2 to protect them from being deacetylated by HDACs until they are deposited into chromatin [122, 153, 154]. Histone H4K16ac supports fork progression and facilitates Fanconi anemia complementation group D2 (FANCD2) recruitment at stalled forks [197, 198]. Histone methylation also plays an important role in S phase. Histone H3.1 is methylated at K9 prior to nucleosome assembly [199]. After incorporation, Suv39 enzymes convert H3.1K9me1 to H3.1K9me3, marking regions of heterochromatin [199]. The decline of H4K20me1 during S phase reflects its licensing role in G1, indicating a transition from origin licensing to active replication [190]. Histone H3K4 methylation (me1, me2, me3) transiently increases after DNA replication, particularly in newly incorporated histones, before resetting to unmodified states—suggesting a replication-specific role [190, 200]. H3K36me3 plays a key role in DNA repair by recruiting BRCA1 and the Rad51 recombinase to promote homologous recombination (HR), the primary repair pathway during S phase [201–203]. Phosphorylation of histone H2A.X by the ATM and ATR kinases generates γH2A.X, which serves as a platform for recruitment of 53BP1 and other DNA damage response proteins [193, 204]. These PTMs reinforce replication-associated repair and maintain fork stability. These dynamic changes in the modification levels depicted in Fig. 4, highlight how acetylation, methylation, ubiquitination, and phosphorylation converge to preserve replication fidelity.

In G2 phase after DNA replication, chromatin modifications ensure genome integrity and prepare the cell for mitosis. In yeast, methylation of H3K79 recruits the nucleotide excision repair factors RAD16 and RAD7 to verify replication accuracy [205]. In higher eukaryotes, changes in acetylation and methylation PTMs balance chromatin accessibility with compaction. Histone H3.1K9me3 reaches its highest levels during S phase, and these levels are maintained in G2 once the replication fork passes through this modification in chromatin [188]. The amount of H4K16ac modifications rise steadily from S into G2, preventing premature chromatin condensation and maintaining DNA accessibility for repair [188, 190]. Figure 4 highlights these transitions in G2, where acetylation maintains open chromatin while methylation prepares chromatin for structural reorganization [206].

Mitosis requires extensive chromatin condensation and checkpoint integration. Phosphorylation of histone H3 at S10 and S28 by the Aurora B kinase is enriched in M phase and drives chromatin compaction [152, 207]. Haspin kinases phosphorylate histone H3 at T3 (H3T3ph), which is recognized by the chromosomal passenger complex containing Aurora B, amplifying phosphorylation levels to promote proper kinetochore function [208]. H4K20me1, which declined in S phase, rises again in mitosis and recruits condensin to compact chromatin [190]. This mark antagonizes H4K16ac, allowing condensation to proceed [190]. Notably, acetylation of histone H4K16 is maintained by lysine acetyltransferase 8/males absent on the first (KAT8/MOF), and is necessary to ensure chromosomal stability for segregation during cell division [59].

The integration of DNA repair and cellular checkpoints continues as mitosis progresses. In late G2/M histone H4K20me3 peaks, recruiting the crumbs homolog 2 (Crb2) checkpoint mediator, which is the *Schizosaccharomyces pombe* ortholog of mammalian 53BP1, to provide a final opportunity for non-homologous end joining (NHEJ) repair before chromosome segregation [209, 210]. Phosphorylation modifications H3S10ph and H3S28ph cooperate with H4K20me3 not only to drive condensation, but also to bookmark persistent DNA lesions, recruiting repair proteins post-mitosis to resolve inherited breaks [211, 212]. These processes, emphasize how phosphorylation and methylation in M phase link chromatin condensation with checkpoint repair functions.

Across the cell cycle, histone modifications function in precise, stage-specific combinations. In G1, acetylation and H4K20me1 establish replication competence and repair priming. In S phase, acetylation, methylation, and phosphorylation stabilize replication forks and facilitate HR repair. In G2, acetylation maintains accessibility while methylation primes chromatin for compaction. In M phase, phosphorylation and methylation orchestrate condensation and checkpoint responses. Figure 4 illustrates how PTM abundance fluctuates across the cellular phases and how combinatorial regulation safeguards genome integrity. The highly dynamic nature of the histone code makes it challenging to understand how it regulates chromatin accessibility, genome organization, and DNA-templated processes including transcription and replication [98, 213]. In the next section, we will provide examples of how disruption of the epigenetic landscape contributes to the development of disease. This knowledge is key for developing strategies to mitigate the harmful effects associated with epigenetic abnormalities.

## The role of histone modifications in human disease

**Fig. 5:**
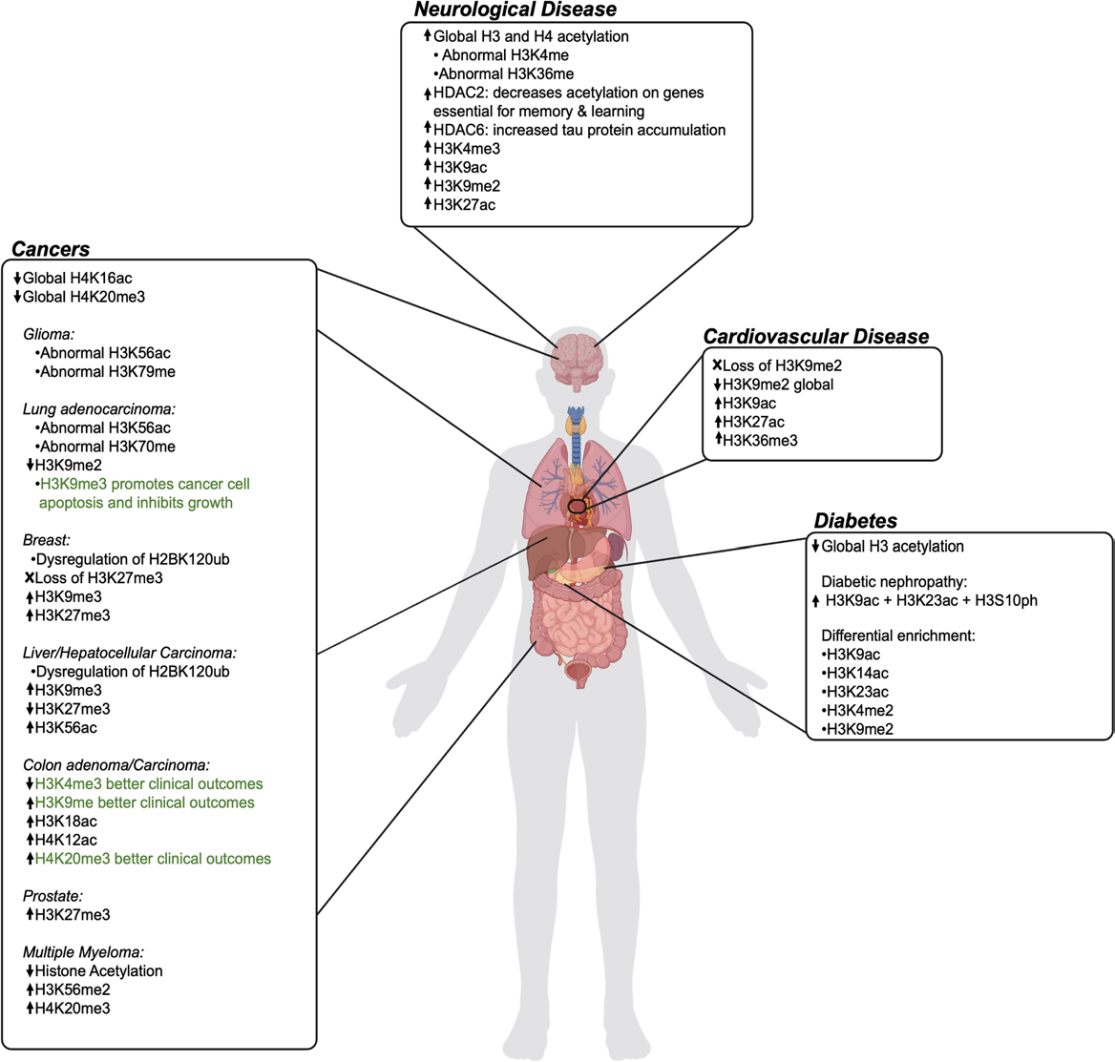
Distribution of histone post-translational modifications in diseases. This figure illustrates various human organs annotated with histone PTMs associated with specific diseases such as cancer, cardiovascular disease, diabetes, and neurological disorders. An upward arrow signifies an increase in the PTM level, while a downward arrow denotes a decrease. PTMs highlighted in green are correlated with favorable clinical outcomes. This figure was adapted from references [217–230]

Over the past decade, there has been an unprecedented amount of research linking epigenetic regulation through histone PTMs to various human diseases. Disruption of these fundamental gene regulatory mechanisms plays a crucial role in initiating and promoting the progression of various diseases, such as cancer, cardiovascular, metabolic, and neurological disease (Fig. 5). Both canonical and variant histone PTMs can alter chromatin structure and regulate nuclear processes related to gene expression, either independently or in combination with other modifications [43, 214, 215]. Dysregulation of PTMs involved in cell cycle regulation are often observed in disease phenotypes, particularly cancer [216]. Over two million cases of cancer were diagnosed in 2025, with more than 618,000 deaths, and many human cancer cells have “epigenetic abnormalities” [217–219]. In cardiovascular disease (CVD), histone modifications influence the complex etiology of cardiovascular pathophysiology, ultimately contributing to disease progression [220–222]. Dysregulated epigenetic modifications are also common in metabolic diseases, including type 2 diabetes, which can cause complications such as kidney disease, neuropathy, and CVD [223–228]. In neurological disorders, including Alzheimer’s disease (AD), epigenetic mechanisms influence gene expression during neurogenesis and neurodevelopment [229, 230].

Despite these strong associations, major challenges remain in linking specific histone PTMs to disease. One key issue is that the transient nature of many PTMs complicate efforts to capture their precise role in disease initiation or progression. Additionally, histone PTMs are not uniformly distributed across the genome, and their context-dependent effects make it difficult to establish causality [231]. Furthermore, studies using bulk tissue samples may mask cell-type-specific epigenetic changes that are critical in disease pathology. Correlative studies are abundant, and our inability to distinguish between the cause and consequence of disease states hinders translational progress [232]. Revealing the mechanistic details of how specific PTMs drive disease phenotypes is paramount for the development of targeted therapeutic strategies. If we can decipher specific meanings of the histone code, we will be able to reverse changes that contribute to phenotypic aberrations and improve patient outcomes.

### Phosphorylation modifications

Dysregulation of histone phosphorylation contributes to disease development as it plays a critical role in DNA repair, chromatin compaction, and transcriptional regulation. Efficient DNA repair is essential for keeping the genome intact, and it prevents the accumulation of mutations that can lead to cancer [204]. Phosphorylation of the histone variant H2A.X at serine 139 (γ-H2A.X) is a well-characterized marker of DNA double-strand breaks, initiating the DNA damage response by recruiting repair proteins to chromatin lesions [233]. Loss or impairment of phosphorylation of histone H2A.X has been linked to the growth and spread of cancer [234, 235]. Additionally, aberrant phosphorylation patterns in cancer frequently co-occur with altered acetylation and methylation modifications, which disrupts the expression of oncogenes and tumor suppressors [236, 237].

In CVD, phosphorylation of histone H3S10 in combination with acetylation of histone H3K14 and H4K8 is associated with oxidative modification of low-density lipoprotein (LDL), a key driver of atherosclerosis and cardiac complications [222]. These epigenetic changes contribute to vascular inflammation and remodeling, underscoring their role in cardiovascular pathology [222].

Histone phosphorylation also plays a role in metabolic disorders. In a type 2 diabetic mouse model, H3S10ph was significantly elevated in cardiac tissue, along with shifts in H3 acetylation and methylation profiles [223]. Phosphorylation of histone H3S10 is typically associated with chromatin compaction during mitosis to promote cell division [238]. The occurrence of this modification in non-dividing cells, may reflect pathological reactivation of cell division pathways, a known contributor to diabetic cardiomyopathy [223].

In Alzheimer’s disease (AD), abnormal cytoplasmic accumulation of histone H3S10ph was observed in neurons, where it colocalized with phosphorylated tau protein, an early marker of AD pathology [239]. These neurons exhibited signs of aberrant cell cycle re-entry, and H3S10ph in the cytoplasm was interpreted as a marker of mitotic catastrophe—an irreversible process leading to neuronal dysfunction and death [239].

Studying histone phosphorylation in the context of disease remains challenging due to their transient nature, as well as the technical limitations in detecting site-specific phosphorylation events occurring in low abundance [240]. The crosstalk between phosphorylation and other modifications further complicates the functional interpretation, especially in cancer and neurodegenerative diseases [241]. Recent advancements in high-resolution mass spectrometry and single-cell multi-omics have enabled more precise mapping of phosphorylation sites and their dynamic changes during disease progression [242, 243]. Additionally, the development of engineered protein binders and clustered regularly interspaced short palindromic repeats (CRISPR)-based epigenome editing tools offers new avenues to dissect the functional roles of histone phosphorylation in vivo [244]. These innovations are helping to overcome previous barriers, paving the way for deeper insights into the role of histone phosphorylation in disease mechanisms. Complementary use of transgenic mouse models and patient-derived cell lines are crucial for investigating the functional significance of histone phosphorylation in disease contexts [245]. These models allow for the manipulation of specific kinases and phosphatases, enabling direct evaluation of how phosphorylation impacts gene expression and disease phenotypes [51].

### Acetylation modifications

Certain types of cancer exhibit translocations that result in abnormal fusion, deletion, or mutations in KAT writer activity, causing an imbalance in histone acetylation levels in chromatin [246, 247]. Interestingly, certain cancer types appear to be associated with specific changes in the prevalence of individual histone modifications. In prostate cancer, the genomic positioning of acetylated and unmodified histone H2A.Z variants was found to regulate gene expression to promote a pro-tumor state [248].

In cardiovascular disease (CVD), the activity of chromatin modifying enzymes that write and erase histone modifications can be altered. For example, the activity of the histone deacetylase Sirtuin 1 (SIRT1), which is known to have a protective effect against CVD, is downregulated after cardiac injury [249]. The levels of SIRT1 are also found to be reduced in coronary artery disease [250]. Increased levels of histone acetylation can also be caused by an up-regulation of KAT activity. For instance, upregulation of p300 is linked to improper left ventricular remodeling, which contributes to the underlying pathological etiology of a heart attack, or myocardial infarction [251].

Diabetes also alters the acetylation state of various genes, and a loss of activity is observed in retinal histone acetyltransferase (HAT) and HDAC activities, resulting in a decrease in global H3 acetylation levels [225]. During hyperglycemia, acetylation can occur at the promoters of inflammatory genes through the activity of the p300 acetyltransferase and NF-kappa B-associated factors. This acetylation is thought to activate the inflammatory genes during high blood sugar periods, and can lead to eventual complications [252].

In neurological disorders dysregulation of histone acetylation is linked to a decline in memory [253, 254]. Increased expression levels of p300/cAMP response element-binding protein (p300/CBP) and TBP-related associated protein part (TRAPP), a component of the Spt-Ada-Gcn5 acetyltransferase (SAGA) complex, was observed in AD brains [255]. These histone acetyltransferase enzymes mediate increased acetylation of histone H3 at K9 and K27, thereby altering gene expression pathways that contribute to AD [255]. An increase in the HAT activity of p300 has also been reported in the hippocampal area of AD patients [256]. In Parkinson’s disease, a global increase in histone H3K27 acetylation is observed, and it is particularly enriched at the promoter regions of disease-related genes [257, 258].

Unlike the rapid and transient nature of phosphorylation modifications, acetylation changes can be more stable and are linked to long-term alterations in gene expression profiles, especially in cancer and neurological disorders [259]. A major challenge in studying histone acetylation in disease is the large number of acetylation sites and the wide range of HATs and HDACs that regulate them. This leads to significant functional redundancy and context-dependent effects [260], making it difficult to pinpoint the specific roles of individual acetylation events. The complexity is further compounded by the interplay between histone acetylation and cellular metabolism, as metabolites can directly modulate acetylation patterns and influence disease progression [261].

Recent technological advances, such as single-cell assay for transposase-accessible chromatin using sequencing (ATAC-seq) and improved quantitative proteomics, have enabled high-resolution analysis of acetylation landscapes, revealing cell-type-specific and disease-associated patterns [262]. The development of small-molecule inhibitors targeting specific HATs and HDACs is opening new therapeutic avenues, although challenges include off-target effects and drug resistance [263]. The integration of these cutting-edge approaches is revealing the complex roles of histone acetylation in disease and informing improved diagnostic and therapeutic strategies [264]. In parallel, transgenic mice and patient-derived organoids are widely used to investigate the role of histone acetylation in disease development and progression [265]. These models enable precise manipulation of HATs and HDACs, offering insights into how altered histone acetylation affects gene expression and cellular function in pathological contexts.

### Methylation modifications

Abnormal methylation patterns on histone H3K4, H3K27, and H3K36 are frequently observed in cancer and vary across tumor types [266–268]. For instance, colorectal cancer tissues often exhibit elevated H3K9me1 levels relative to normal colonic mucosa [266, 269, 270]. Interestingly, patients with colon cancers characterized by high levels of H3K9me3 and H4K20me3, combined with reduced H3K4me3, tend to have better clinical outcomes [271]. hypermethylation of H3K79 is driven by overexpression of the methyltransferase DOT1L (disruptor of telomeric silencing 1-like), leading to increased transcription of MLL target genes [272]. DOT1L is also upregulated in breast cancer. Inhibition of DOT1L reduces H3K79 methylation, downregulates genes associated with breast cancer progression, and suppresses the epithelial-to-mesenchymal transition (EMT), which is a hallmark of tumor metastasis [273]. Additional examples of methylation-driven oncogenesis include overexpression of the jumonji domain-containing protein 2 A (JMJD2A) demethylase, which decreases H3K9me3 and H3K36me3 levels, contributing to colorectal tumorigenesis [274]. In lung cancer, reduced expression of the G9a methyltransferase correlates with decreased H3K9me2, further promoting malignant progression [275].

Histone methylation also plays a critical role in cardiovascular pathology. In models of pathological cardiac hypertrophy, a loss of H3K9me2 leads to the reactivation of fetal gene programs and abnormal cardiomyocyte growth, weakening cardiac function [276, 277]. A global reduction in the histone H3K27me3 mark has been observed in blood vessels and smooth muscle cells associated with atherosclerotic plaques [278, 279]. In cardiomyopathy, a genome wide analysis revealed enrichment of H3K36me3 in failing human hearts, implicating this mark in maladaptive gene expression responses [276].

In diabetes patients, aberrant methylation patterns contribute to gene silencing and oxidative stress. The superoxide dismutase-2 (SOD2) enzyme functions to protect cells from oxidative stress [280]. In retinal tissue from diabetic models, high levels of H4K20me3, H3K9ac, and the suppressor of variegation 4–20 homolog 2 (SUV420h2) methyltransferase were found at the promoter and enhancer regions of the *SOD2* gene [281]. Together, they recruit lysine demethylase-1 (LSD1), which demethylates histone H3K4 at the *SOD2* promoter, resulting in *SOD2* repression [282]. Even after glucose normalization, levels of histone H4K20me3 and H4K9ac remain elevated, suggesting a lasting epigenetic imprint that may perpetuate oxidative damage in diabetic retinopathy [281].

Aberrant histone methylation has also been implicated in neurodegenerative disorders. In AD patients increased levels of H3K4me3 are detected in affected brain regions, likely reflecting the hyperactivation of methyltransferases [283, 284]. In Parkinson’s disease, histone H3K4me3 is enriched at the promoter regions of the *SCNA* gene in patient brains. This gene encodes for α-synucleinin, which contributes to neuron degeneration, and the levels of H3K4me3 positively correlates to increased expression of the α-synucleinin protein [285]. Collectively, these findings suggest that aberrant histone methylation, particularly H3K4me3 enrichment, plays a critical role in epigenetic mis-regulation driving neurodegenerative disease, underscoring the need for deeper mechanistic studies to clarify its causal impact.

Deciphering the role of histone methylation in disease remains challenging, as the same modification can exert opposing effects depending on its genomic context and the presence or absence of other histone marks [286]. Adding to the complexity, histone methyltransferases (HMTs) and lysine demethylases (KDMs), which often have overlapping substrate specificities, are frequently mutated or dysregulated in cancer and developmental disorders, disrupting gene expression programs and promoting aberrant cellular behavior [287].

Recent advances in single-molecule and single-cell technologies, along with integrative multi-omics approaches, have begun to unravel the complex regulatory networks governed by histone methylation, identifying novel regulatory mechanisms and therapeutic targets [288]. Locus-specific manipulation of methyl marks using CRISPR-dCas9-based epigenome editing tools now allows researchers to directly test the functional relevance of specific methylation events [289]. In parallel, high-throughput ChIP-seq and mass spectrometry-based proteomics are shedding light on methylation dynamics and PTM crosstalk [290]. Animal models and patient-derived organoids have been instrumental in validating the roles of specific HMTs and KDMs in disease and are critical platforms for testing methylation-targeted therapeutics [245, 291].

### Ubiquitination modifications

Histone ubiquitination plays a central role in the endothelial to mesenchymal transition (EMT), which is a major factor in cancer progression [292]. The snail family transcriptional repressor 1 (SNAI1/Snail) protein is a key transcriptional repressor that is known to stimulate EMT [292]. SNAI1 interacts with the E3 ubiquitin ligases ring finger protein 1 A (Ring1A), and its paralog Ring1B, to form a regulatory complex that promotes mono-ubiquitination of histone H2A at lysine 119 (H2AK119ub) [292]. This modification leads to transcriptional repression of target genes and the induction of EMT, enhancing tumor invasiveness and metastatic potential [292]. The same SNAI1-Ring1A/B complex has also been implicated in CVD. By inducing EMT in vascular endothelial cells, it promotes the formation of atherosclerotic lesions [293]. This highlights a shared epigenetic pathway that drives both oncogenesis and vascular pathology.

Long-term hyperglycemia is associated with altered histone ubiquitination patterns that contribute to diabetic complications [294]. In diabetic patients, increased H2AK119ub and decreased H2BK120ub levels were observed in peripheral blood cells of individuals with high glucose levels compared to those receiving glucose-lowering interventions [294]. These differences in histone ubiquitination levels was linked to changes in transforming growth factor-beta (TGF-beta) expression, which is a major player in the development of kidney disease. This study concluded that elevated histone H2AK119ub may promote the development of diabetic nephropathy, while histone H2BK120ub may exert a protective role in the development and progression of disease [294].

In Alzheimer’s Disease downregulation of BMI1(B‐cell‐specific Moloney murine leukemia virus integration site 1) has been linked to neurodegeneration. BMI1 is an E3-ubiquitin ligase that typically functions as part of a complex with Ring1A and Ring1B, catalyzing the formation of H2AK119ub to repress developmental genes and maintain chromatin compaction [295]. Loss of BMI1 in the brains of AD patients leads to decreased H2AK119ub, resulting in aberrant gene expression and chromatin decompaction that may accelerate neurodegenerative processes [295].

Unlike other histone modifications, ubiquitination often serves as a signal for protein–protein interactions, directly influencing the recruitment of DNA repair and transcriptional machinery, which makes elucidating its role disease particularly complex [296]. Another challenge is the transient and low-abundance nature of ubiquitinated histones, which hampers their detection and quantification in clinical samples [297]. Additionally, the interplay between ubiquitination and the proteasome pathway further complicates analysis, as histone ubiquitination can tag proteins for degradation or modulate their function without degradation [298].

Technological innovations, such as ubiquitin remnant profiling and high-resolution mass spectrometry, are enabling more comprehensive mapping of ubiquitination sites and their disease-associated dynamics [299]. Transgenic mouse models and patient-derived organoids are increasingly being used to functionally characterize E3 ubiquitin ligases and deubiquitinases, which has revealed new therapeutic targets. Still, off-target effects and the pleiotropic roles of ubiquitination enzymes pose significant challenges [300].

## Translating the histone code into clinical insight

Histone post-translational modifications regulate chromatin accessibility and gene expression, providing a dynamic and reversible layer of epigenetic control [301]. More than 300 histone PTMs have been described, and over 200 histone proteoforms have been characterized, reflecting the scale and combinatorial complexity of this network [117, 123]. Each proteoform represents a unique molecular variant of a histone carrying a specific set of modifications, and these combinations integrate metabolic, signaling, and developmental cues to fine-tune chromatin function. Dysregulation of PTM patterns is now firmly associated with major human diseases, including cancer, cardiovascular disease, metabolic syndromes, and neurodegenerative disorders.

The reversibility of histone modifications makes them attractive therapeutic targets. Small-molecule inhibitors against epigenetic enzymes, including histone acetyltransferases (HATs), histone deacetylases (HDACs), histone methyltransferases (HMTs), lysine demethylases (KDMs), and ubiquitin ligases, are in various stages of clinical development [222, 229, 263, 264, 302]. Several HDAC inhibitors, such as vorinostat and romidepsin, are FDA-approved for cutaneous T-cell lymphoma, while bromodomain inhibitors targeting bromodomain and extra-terminal domain (BET) proteins are in advanced clinical trials for leukemia and solid tumors [303, 304]. BET inhibitors have a unique mechanism of action compared to existing drugs and have been shown to produce anti-proliferative activity across a range of tumor types [303, 305]. Trotabresib is a next-generation BET inhibitor that provides potent and selective BRD inhibition, has antitumor activity in patients with advanced solid tumors, and demonstrates a tolerable long-term safety profile [303].

Clinical research has also begun to explore combination therapies, in which PTM-targeting drugs are paired with DNA methyltransferase inhibitors such as azacytidine, reflecting the interplay between histone modifications and DNA methylation [306–309]. The efficacy of these drugs, however, depends on understanding how PTM networks operate in a temporal, spatial, and combinatorial manner. Inhibiting a single enzyme may have broad, context-dependent effects depending on which PTMs coexist in a given chromatin environment.

Beyond therapeutic targeting, histone modifications have potential as biomarkers. Specific PTM patterns may reflect disease onset, severity, or treatment response. For instance, altered levels of H3K27me3 and H3K9me3 have been correlated with tumor aggressiveness, while global histone acetylation states are linked to prognosis in cardiovascular and metabolic disease [310–312]. Although preliminary studies suggest that PTM patterns could serve as diagnostic or prognostic markers [123, 240, 313–315], the field lacks standardized approaches for measuring and interpreting these signatures across diverse patient populations.

## Future directions

Future research must address these gaps with integrative, scalable, and high-resolution strategies. Single-cell PTM profiling is beginning to reveal how histone modifications vary between individual cells across tissues and within tumors, providing insights into cellular plasticity and tumor heterogeneity [316, 317]. Top-down mass spectrometry now allows direct characterization of intact histone proteoforms, capturing the full complement of modifications on a single histone molecule [117, 123, 124, 318, 319]. These proteomic advances, combined with functional genomic assays such as chromatin immunoprecipitation sequencing (ChIP-seq), ATAC-seq, and RNA-sequencing, will clarify how histone PTMs intersect with DNA methylation, chromatin remodeling, and non-coding RNAs to regulate DNA accessibility and gene expression. For example, the newly developed Cleavage Under Targets & Tagmentation (CUT&Tag) technique is an alternative chromatin profiling strategy to the classical ChIP-seq and yields a higher efficiency [320]. CUT&Tag uses primary and secondary antibody binding to chromatin proteins and modifications to profile single cell chromatin [320]. However, ChIP-seq is not practical for the small material volume characteristic of patient samples [320, 321]. A variant of CUT&Tag known as CUTAC (Cleavage Under Targeted Accessible Chromatin), builds from ATAC-seq and CUT&Tag and empowers affordable epigenomic profiling for clinical applications and biomarker identification [321].

Computational approaches, including machine learning and artificial intelligence, will be essential for interpreting these complex datasets [322]. Predictive models trained on multi-omics data can uncover hidden relationships between PTM signatures and cellular outcomes, and may eventually guide therapeutic decision-making [322]. Collaborative consortia and data-sharing networks between research scientists and clinicians from diverse disciplines will also be critical, ensuring that findings are reproducible and broadly applicable across tissues, disease states, and patient populations [323].

Together, these efforts will advance the translation of the histone code from a conceptual framework into a clinical tool. By defining how PTM combinations specify biological outcomes, researchers can develop both targeted therapies and robust biomarkers. This convergence of mechanistic biology, proteomics, and computational modeling has the potential to transform histone modifications into actionable information for precision medicine.

## Supplementary Information

Additional file 1: Figure S1. Common names of biologically-relevant post-translational modifications and their chemical structures. Tables S1a-S1d. Post-translational modifications present on the N-terminal tails of histone proteins. Tables S1a-S1d list histone post-translational modifications (PTMs) that are mainly present on the N-terminal tail region of histone proteins, and are organized by modification. The table includes the histone, which is color-coded in the canonical colors (H3: blue, H4: green, H2A: yellow, H2B: red), the location of the modification (Location) with the extent of the methylation modification (in Table S1c), the proposed function, and associated references (References). Table S2. Histone mutations that phenocopy deletion of the associated writer enzyme. Figure S2. Interplay of histone modifications on the binding activity of the ATAD2 and ATAD2B bromodomains. The bromodomains of ATAD2 and ATAD2B preferentially bind di-acetylated H4K5acK12ac over other di-acetyllysine combinations such as H4K5acK8ac [153]. b. The presence of adjacent PTMs with a different shape, size, and charge, located close to an acetylated lysine residue can have a significant impact on bromodomain binding activity. Di-methylation of Arg 3 on histone H4 considerably decreases the binding affinity of the ATAD2 bromodomain for the H4K5ac modification from 58 μM to 211 μM (orange dashed line). However, the ATAD2B bromodomain still binds to H4R3me2aK5ac robustly (green check mark and arrow). c. The ATAD2B bromodomain shows strong affinity for the acetylated histone variants H2A.XK5ac and H2A.ZK4ac (green check mark and arrow), while the ATAD2 bromodomain preferentially interacts with the acetylated version of canonical histone H2AK5ac. The ATAD2 bromodomain binds much weaker to histone H2A.XK5ac than the ATAD2B bromodomain (orange slash and dashed line), and does not interact with acetylated histone H2A.Z ligands (depicted as a red X) [153, 324–362].

## Data Availability

No datasets were generated or analysed during the current study.
