# Supplementary material for: Unraveling the complexity of the histone code: implications for gene regulation and disease
Source: Genome Biol. 2026 Mar 24;27:150. doi: 10.1186/s13059-026-04011-3 (PMC13134275; doi:10.1186/s13059-026-04011-3)
Supplement: Supplementary file 1 — Additional file 1: Figure S1. Common names of biologically-relevant post-translational modifications and their chemical structures. Tables S1a-S1d. Post-translational modifications present on the N-terminal tails of histone proteins. Tables S1a-S1d list histone post-translational modifications (PTMs) that are mainly present on the N-terminal tail region of histone proteins, and are organized by modification. The table includes the histone, which is color-coded in the canonical colors (H3: blue, H4: green, H2A: yellow, H2B: red), the location of the modification (Location) with the extent of the methylation modification (in Table S1c), the proposed function, and associated references (References). Table S2. Histone mutations that phenocopy deletion of the associated writer enzyme. Figure S2. Interplay of histone modifications on the binding activity of the ATAD2 and ATAD2B bromodomains. The bromodomains of ATAD2 and ATAD2B preferentially bind di-acetylated H4K5acK12ac over other di-acetyllysine combinations such as H4K5acK8ac [153]. b. The presence of adjacent PTMs with a different shape, size, and charge, located close to an acetylated lysine residue can have a significant impact on bromodomain binding activity. Di-methylation of Arg 3 on histone H4 considerably decreases the binding affinity of the ATAD2 bromodomain for the H4K5ac modification from 58 μM to 211 μM (orange dashed line). However, the ATAD2B bromodomain still binds to H4R3me2aK5ac robustly (green check mark and arrow). c. The ATAD2B bromodomain shows strong affinity for the acetylated histone variants H2A.XK5ac and H2A.ZK4ac (green check mark and arrow), while the ATAD2 bromodomain preferentially interacts with the acetylated version of canonical histone H2AK5ac. The ATAD2 bromodomain binds much weaker to histone H2A.XK5ac than the ATAD2B bromodomain (orange slash and dashed line), and does not interact with acetylated histone H2A.Z ligands (depicted as a red X) [153, 324–362]. [file 13059_2026_4011_MOESM1_ESM.pdf]

Supplementary Materials

Unraveling the complexity of the histone code: implications for gene regulation and disease

Kiera L. Malone[1], Ajit K. Singh[1], James M. Lignos[1], Elizabeth D. Cook[1,2,3], Margaret Phillips[1], Brian W. Boyle[1,2], Isabelle A. Kressy[1,2], Mirabella Vulikh[1,2], Annika K. Lathrop[1,2], Kyle T. McKay[1], Hassan Zafar[1], Janet L. Stein[2,3], Gary S. Stein[2,3], and Karen C. Glass[1,2,3*]

[1]Department of Pharmacology, Larner College of Medicine, University of Vermont, Burlington, VT, 05405, USA
[2]Department of Biochemistry, Larner College of Medicine, University of Vermont, Burlington, VT, 05405, USA
[3]University of Vermont Cancer Center, University of Vermont, Burlington, VT 05405, USA.

* To whom correspondence should be addressed. Tel: +1(802) 656-5760; Fax: +1(802) 656-4523; Email: karen.glass@med.uvm.edu

Table of Contents

Figure S1: Common names of biologically-relevant post-translational modifications and their chemical structures……………………………………………………….……………………..3
Table S1a: Phosphorylation PTMs found on histone proteins and their proposed function……....4
Table S1b: Acetylation PTMs found on histone proteins and their proposed function………..…..6
Table S1c: Methylation PTMs found on histone proteins and their proposed function……….....9
Table S1d: Ubiquitination PTMs found on histone proteins and their proposed function……....14
Table S2: Histone mutations that phenocopy deletion of the associated writer enzyme……..…..15
Figure S2: Interplay of histone modifications on the binding activity of the ATAD2 and ATAD2B bromodomains……………………………...……………………………………….……16

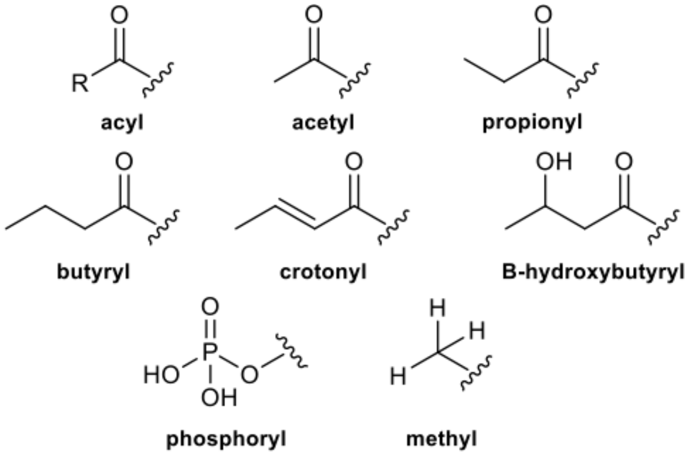

Figure S1. Common names of biologically-relevant post-translational modifications and their chemical structures.

Tables S1a-S1d: Post-translational modifications present on the N-terminal tails of histone proteins. Tables S1a-d list histone post-translational modifications (PTMs) that are mainly present on the N-terminal tail region of histone proteins, and are organized by modification. The table includes the histone, which is color-coded in the canonical colors (H3: blue, H4: green, H2A: yellow, H2B: red), the location of the modification (Location) with the extent of the methylation modification (in Table S1c), the proposed function, and associated references (References).

**Table S1a: Phosphorylation PTMs found on histone proteins and their proposed function**			
**Histone**	**Location**	**Proposed function**	**References**
H3	T3	• **Cell cycle regulation**<br>○ Ensures proper chromosome separation for cell division	(38)
H3	T6	• **Transcriptional regulation**<br>○ Controls the level of H3K4 methylation by preventing the histone lysine demethylase 1-mediated removal of the active mono- and di-methylation marks	(38)
H3	S10	• **Gene expression activation**<br>○ Allows for chromatin to undergo decondensation<br>▪ Initiates or prevent the recruitment of chromatin binding proteins to prepare for active gene expression and cell cycle progression	(324, 325)
H3	T11	• **Transcriptional activation**<br>○ Causes targeted de-methylation of histone residues that prevent active gene expression	(326)
H3	S28	• **Transcriptional activation**<br>○ Works together with acetylated residues to ensure proper conditions<br>• **Stress response**<br>○ Prevents deacetylation of histone residues necessary for activation of the stress response at promoters	(38, 47)
H3	Y41	• **Important for:**<br>○ Histone turnover<br>○ Chromatin architecture	(327, 328)
H3	Y99	• **Important for:**<br>○ Histone turnover<br>○ Chromatin architecture	(327, 328)
H3	T45	• Facilitates termination of gene transcription in response to DNA damage	(184)
H4	S1	• Involved in DNA damage response<br>• Regulates chromatin structure and gene expression	(46, 74)

		• Promotes nucleosome reorganization of histone variants	
H4	S47	• Involved in DNA damage response<br>• Regulates chromatin structure and gene expression<br>• Promotes nucleosome reorganization of histone variants	(46, 74)
H2A	T120	• **Transcription regulation**<br>• Required for DNA topoisomerase II alpha to localize to the centromeric region	(48)
H2A	T133	• Connected to centromere maintenance and operation during meiosis	(329)
H2A	S139 (gamma-H2A.X)	• DNA damage response and repair processes	(330)
H2B	S14	• **DNA damage response**<br>• Colocalizes with markers of DNA double-strand breaks and observed following damage by ionizing radiation	(331)
H2B	S36	• Regulation of gene expression	(332)
H2B	Y37	• Suppresses expression of replication-dependent core histone genes	(49)

Table S1b: Acetylation PTMs found on histone proteins and their proposed function			
**Histone**	**Location**	**Proposed function**	**References**
H3	K4	• **Transcriptional activation**<br>○ Extends into the coding regions on actively transcribing genes<br>• Present on active genes in the promoter and transcribed regions	(62, 333)<br>(75)
H3	K9	• **Transcriptional regulation**<br>○ Recruits the super elongation complex (SEC) to the nucleosome, helping to regulate transcription from initiation to elongation<br>• Present in the region around the transcription start site	(56, 62)<br>(75)
H3	K14	• **Transcriptional activation**<br>○ Found enriched over promoters, introns, intergenic regions, and gene bodies in Drosophila embryos<br>▪ Bound by a bromodomain in the Brahma protein, a subunit of Switch/Sucrose Non-Fermentable (SWI/SNF) chromatin remodeling complex, which is responsible for proper wing formation<br>• **DNA Damage response**<br>• Recruits a chromatin remodeling complex via its bromodomain (RSC: Remodels the Structure of Chromatin) in yeast to "nucleosome barriers" to facilitate the repair of UV damaged-induced DNA double strand breaks	(57, 75, 334)
H3	K18	• **Transcriptional regulation**<br>○ Recruits the co-activator associated arginine methyltransferase 1 (CARM1) to methylate H3R17 for gene activation<br>• Present in the region around the transcription start site	(62, 75, 335, 336)
H3	K23	• **Transcriptional activation**<br>○ Recruits CARM1 to methylate H3R17 for gene activation<br>• Positive correlation with gene expression levels	(75, 336, 337)
H3	K27	• Enrichment at active enhancer regions on a genome-wide scale in human HeLa cells<br>• Present in the region around the transcription start site (TSS)	(62, 338)
H3	K36	• **DNA damage response**	(62, 201)

		◦ After acetylation by the GCN5 histone acetyltransferase in yeast, this PTM promotes open chromatin to increase the rate of histone exchange on DNA to facilitate homologous recombination<br>• Mainly present in the region around the TSS	
H3	K56	• **Transcriptional activation**<br>◦ Found during transcriptionally active conditions at the yeast *GAL1* promoter<br>◦ Necessary for proper positioning of RNA polymerase II to ensure transcription initiation	(75, 339) (340)
H4	K5	• **Cell cycle regulation**<br>◦ Marks found on newly synthesized histones that ensure their proper deposition<br>◦ Important for progression through the cell cycle<br>• Present on active genes in the promoter and transcribed regions	(62, 120, 341, 342)
H4	K8	• **Transcriptional activation**<br>◦ Present in active genes at the promoter and transcribed regions<br>• Ensures chromatin decompaction for proper DNA replication in yeast	(62, 75) (343)
H4	K12	• **Cell cycle regulation**<br>◦ Marks found on newly synthesized histones that ensure their proper deposition<br>◦ Important for progression through the cell cycle<br>• Present on active genes in the promoter and transcribed regions	(120, 341, 342, 344)
H4	K16	• **Transcriptional activation**<br>◦ Present on active genes in the promoter and transcribed regions<br>◦ Found enriched over gene bodies<br>◦ Simulates the Disruptor of Telomeric silencing 1 (DOT1) H3K79 methyltransferase complex to prevent the binding of repressor proteins	(62, 334, 345)
H4	K20	• **Transcriptional repression**<br>◦ Found across the genome in regions where activators are excluded<br>◦ Recruits the neuron-restrictive silencing factor/ repressor element-1 (NRSF/REST), a transcriptional repressor	(346)

H2A	K5	• **Transcriptional regulation**<br>○ Acetylation mark present along gene bodies, also present around predicted promoters in the genome	(75, 347)
H2A	K7	• **Transcriptional activation**<br>○ Found during transcriptionally active conditions at the yeast *GAL1* promoter	(75, 339)
H2A	K9	• Mainly present in the region around the transcription start site	(62)
H2B	K5	• **Transcriptional activation**<br>○ Member of histone H2B N-terminus multisite acetylation (H2BNTac), which is a signature of active enhancers for transcriptional regulation<br>• Signal overlaps with active H3K27ac, and is present in the region around the TSS	(62, 75, 348)
H2B	K12	• **Transcriptional activation**<br>○ Member of histone H2B N-terminus multisite acetylation (H2BNTac), which is a signature of active enhancers for transcriptional regulation<br>• Present on active genes in the promoter and transcribed regions	(62, 348)
H2B	K16	• **Transcriptional activation**<br>○ Member of histone H2B N-terminus multisite acetylation (H2BNTac), which is a signature of active enhancers for transcriptional regulation	(348)
H2B	K17	• **Transcriptional activation**<br>○ Found during transcriptionally active conditions at the yeast *GAL1* promoter	(339)
H2B	K20	• **Transcriptional activation**<br>○ Member of histone H2B N-terminus multisite acetylation (H2BNTac), which is a signature of active enhancers for transcriptional regulation<br>• Present on active genes in the promoter and transcribed regions	(62, 348)
H2B	K120	• **Transcriptional regulation**<br>○ Enriched mark across gene bodies, also present around predicted promoters in the genome<br>• Present on active genes in the promoter and transcribed regions	(62, 347)

Table S1c: Methylation PTMs found on histone proteins and their proposed function			
**Histone**	**Location**	**Proposed function**	**References**
H3	R2me1	• Associated with genes that are highly expressed or medium expressed at sub-telomeric genes	(349)
H3	R2me2a	• **Transcriptional repression**<br>○ Found at regions of heterochromatin in yeast, responsible for silencing<br>○ Found at the least active genes in euchromatin near the middle of the coding region	(350)
H3	R2me2s	• **Transcriptional activation**<br>○ Found at promoters upstream of the TSS and is located near other activation marks that are enhanced at enhancers	(351, 352)
H3	K4me1	• **Transcriptional activation**<br>○ Found on gene coding regions<br>○ Present within transcribed regions of genes and promoters	(127)
H3	K4me2	• **Transcriptional activation**<br>○ Found on gene coding regions<br>○ Found on active genes within transcribed regions in chicken embryo erythrocytes	(61, 353)
H3	K4me3	• **Transcriptional activation**<br>○ Known as a hallmark of the TSS<br>○ Found on active genes within transcribed regions in chicken embryo erythrocytes	(61, 353)
H3	R8me-	• **Transcriptional repression**<br>○ Methylated by Protein Arginine Methyltransferase 5 (PRMT5) to repress gene expression	(84)
H3	K9me1	• **Transcriptional regulation**<br>○ Marker of active genes and is located downstream of the TSS in transcribed regions, acting as the transcriptional initiation site<br>• **Binding platform for gene expression regulation**<br>○ Binding partner for the methyltransferase that forms K9me2/3, making it an important mark in the establishment of heterochromatin	(61, 82)
H3	K9me2,3	• **Transcriptional repression & Heterochromatin formation**<br>○ Important and necessary marks in heterochromatin maintenance<br>○ Present in high levels in silent genes	(61, 82)
H3	R17me1-2a	• **Transcriptional activation**	(83)

		○ Associated with activation of the estrogen-regulated protein (pS2, protein Secreted 2/TreFoil Factor 1)	
H3	K27me1	• **Transcriptional activation & actively expressing genes**<br>○ Found in high levels at active promoters downstream of the TSS<br>○ Associated with high levels of expression. Examples include expression of Signal Transducer and Activator of Transcription 1 and 4 (STAT1 and STAT4).<br>○ Often positioned at gene bodies with high transcriptional activity, and is regulated by the polycomb repressive complex 2 (PRC2)	(61, 81)
H3	K27me2	• **Transcriptional repression**<br>○ Repressive mark involved in gene expression and is involved in gene silencing activities with H3K27me3<br>○ Found in intergenic regions in genes with low transcriptional rates	(62, 81)
H3	K27me3	• **Transcriptional repression**<br>○ Repressive mark involved in gene expression and is involved in gene silencing activities with H3K27me2<br>○ Found at the promoters of silent and repressed genes<br>• **Gene repression and silencing regulation**<br>○ Its presence may impact the modification of other histone residues that are required for transcriptional activation	(62, 81, 354)
H3	K36me1	• **Transcriptional activation**<br>○ May be found in regions toward active promoters across the genome	(61)
H3	K36me2	• **Gene expression activation regulation**<br>○ Found in high levels close to the TSS<br>○ Prevents transcriptional repression by inhibiting PRC2 binding to H3K27me1, preventing the formation of the H3K27me2/3 repressive marks	(355)
H3	K36me3	• **Gene expression activation regulation**<br>○ Found enriched in gene bodies and is present in high levels on active genes in the transcribed regions<br>○ Prevents transcriptional repression by inhibiting PRC2 binding to H3K27me1,	(61, 81, 355)

		preventing the formation of the H3K27me2/3 repressive marks	
H3	R42me2a	• **Transcriptional activation**<br>o Found internally on histones in the nucleosome in an important region where it may be responsible for destabilizing the histone: DNA contacts in preparation for transcriptional activation	(85)
H3	K56me1	• **Transcriptional repression**<br>o Found in regions of the genome excluded from transcription, near heterochromatin<br>• **Cell cycle progression**<br>o Interacts tightly with DNA replication factor Proliferating Cell Nuclear Antigen (PCNA) during G1, which then dissipates as the cells enter S phase, ensuring progression through the cell cycle	(356)
H3	K56me3	• **Heterochromatin regulation**<br>o Found at the chromocenter of heterochromatin in metazoans	(357)
H3	K64me3	• **Transcriptional repression**<br>o Repressive mark found at pericentric heterochromatin near a few promoters<br>o Levels can decrease when chromatin is being remodeled	(358)
H3	K79me1	• No preference for active or repressed genes	(61)
H3	K79me2	• **Gene expression regulation**<br>o Found in the promoters and in open reading frames of expressed genes, potentially correlating with high levels of gene expression<br>• **Cell cycle progression**<br>o Levels change during the cell cycle, increasing through S phase, peaking at G2/M, and decreasing after mitosis, suggesting it regulates progression through the cell cycle	(359, 360)
H3	K79me3	• **Gene expression regulation**<br>o Found in the protein coding regions of expressed genes, potentially correlating with high levels of gene expression<br>o High levels present at the promoters of inactive genes<br>• **Transcriptional silencing regulation**<br>o H3K79me3 and its methyltransferase enzyme, DOT1, prevent a silencing complex, SIR, from binding to chromatin	(61, 359, 360)

H4	R3me2a	• **Gene expression activation**<br>○ Catalyzed by the Protein Arginine Methyltransferase 1 (PRMT1)<br>○ Associated with gene expression activation in human MCF7 cells, enriched at specific super-enhancers, promoters, enhancers<br>○ Activates histone lysine acetyltransferases (HATs)	(87)
H4	R3me2s	• **Gene expression silencing**<br>○ Catalyzed by the Protein Arginine Methyltransferase 5 (PRMT5)<br>○ Found as a repressive modification at the globin genes<br>○ Recruits DNA Methyltransferase 3A (DNMT3A)	(88)
H4	K8me1	• **Stress response**<br>○ Mono-methylated by Set1 in yeast, which may be able to regulate and signal for remodeling complexes in response to stress	(361)
H4	K12me1	• **Stress response**<br>○ Mono-methylated by Set1 in yeast, which may be able to regulate and signal for remodeling complexes in response to stress	(361)
H4	K20me1	• **Binding platform for regulation of gene expression and DNA repair**<br>○ Binding partner for the methyltransferase that forms K20me2/3, making it an important mark in the establishment of their functions<br>• **Cell cycle regulation**<br>○ Levels change and ensure proper progression through the cell cycle	(362)
H4	K20me2	• **DNA damage response**<br>○ Serves as a binding site for a damage repair-associated protein	(362)
H4	K20me3	• **Gene silencing regulation**<br>○ Heterochromatin mark in pericentric regions for silencing and maintenance of cell homeostasis<br>○ Located in silenced heterochromatic regions of the genome<br>• **DNA damage response/stress response regulation**<br>○ Coordinates cell cycle regulation in response to the stress induced after a DNA damage event by having the cells exit G1 phase and enter G0	(362)
H2B	K5me1	• **Associated with actively transcribed genes**	(61)

		• An activation mark located downstream of the TSS and associated with active promoters and gene activation	

**Table S1d: Ubiquitination PTMs found on histone proteins and their proposed function**			
**Histone**	**Location**	**Proposed function**	**References**
H2A	K13	• **DNA damage response**<br>○ Is ubiquitinated by the RING finger protein 168 (RNF168), an E3 ligase, in response to double-strand DNA breaks	(92)
H2A	K15	• **DNA damage response**<br>○ Is ubiquitinated by the RING finger protein 168 (RNF168), an E3 ligase, in response to double-strand DNA breaks	(92)
H2A	K63	• In response to double-strand DNA breaks	(94)
H2A	K119	• Is ubiquitinated by the PRC1 complex in response to UV damage	(93)
H2B	K34	• **Transcriptional activation**<br>○ Ubiquitination may be able to unwrap the DNA surrounding the nucleosomes to assist in activation/elongation	(95)
H2B	K120	• Important for:<br>○ Increased transcription<br>○ DNA replication<br>○ Mitosis<br>○ Meiosis	(91)
H2B	K123	• Responsible for telomeric silencing through interaction with other methylated PTMs	(129)

Table S2: Histone mutations that phenocopy deletion of the associated writer enzyme.			
**Mutation**	**Model/system**	**Functional consequence**	**Phenotypic outcome**
H3K27A(102)	Drosophila	Loss of Polycomb repressive complex 2 (PRC2)-mediated repression	Homeotic transformations, phenocopies PRC2 deficiency
H3K27R(103)	Mouse embryonic stem cells (mESCs)	Prevents PRC2 methylation and CBP/EP300 acetylation	Transcription and differentiation similar to PRC2-null mutants
H3.3K36M(104)	Chondroblastomas	Inhibits SETD2 recruitment, reduces H3K36 methylation	Mimics SETD2 deletion, alters epigenome and cell phenotype

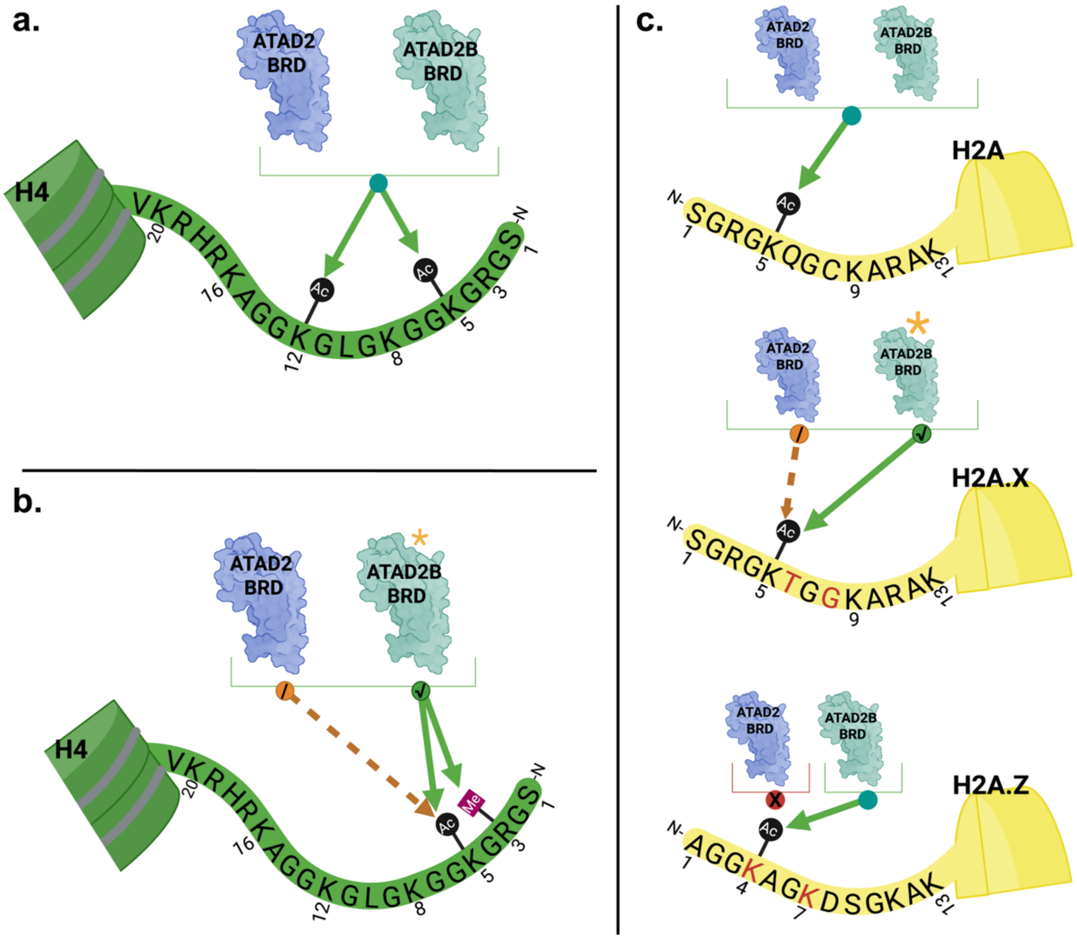

Figure S2: Interplay of histone modifications on the binding activity of the ATAD2 and ATAD2B bromodomains. a. The bromodomains of ATAD2 and ATAD2B preferentially bind di-acetylated H4K5acK12ac over other di-acetyllysine combinations such as H4K5acK8ac(153). b. The presence of adjacent PTMs with a different shape, size, and charge, located close to an acetylated lysine residue can have a significant impact on bromodomain binding activity. Di-methylation of Arg 3 on histone H4 considerably decreases the binding affinity of the ATAD2 bromodomain for the H4K5ac modification from 58 µM to 211 µM (orange dashed line). However, the ATAD2B bromodomain still binds to H4R3me2aK5ac robustly (green check mark and arrow). c. The ATAD2B bromodomain shows strong affinity for the acetylated histone variants H2A.XK5ac and H2A.ZK4ac (green check mark and arrow), while the ATAD2 bromodomain preferentially interacts with the acetylated version of canonical histone H2AK5ac. The ATAD2 bromodomain binds much weaker to histone H2A.XK5ac than the ATAD2B bromodomain (orange slash and dashed line), and does not interact with acetylated histone H2A.Z ligands (depicted as a red X)(153).
